# Crystal Growth Modulation of Tin–Lead Halide Perovskites via Chaotropic Agent

**DOI:** 10.1021/jacs.5c05772

**Published:** 2025-08-23

**Authors:** Yueyao Dong, Wen-Xian Zhu, Dong-Tai Wu, Xuan Li, Robert J. E. Westbrook, Chi-Jing Huang, Zeyin Min, Weiying Hong, Boyuan Wang, Ganghong Min, Sanjayan Sathasivam, Matteo Palma, Stoichko Dimitrov, Chieh-Ting Lin, Thomas J. Macdonald

**Affiliations:** † Department of Electronic & Electrical Engineering, 4919 University College London, Roberts Building, London WC1E 7JE, U.K.; ‡ Department of Chemical Engineering, 34916 National Chung Hsing University, Taichung 40227, Taiwan; § Innovation and Development Center of Sustainable Agriculture, National Chung Hsing University, Taichung 40227, Taiwan; ∥ Department of Chemistry, 4617 Queen Mary University of London, London E1 4NS, U.K.; ⊥ Department of Chemistry, University of Washington, Seattle, Washington 98195, United States; # School of Engineering & Design and Energy, Materials & Environment Research Centre, 4914 London South Bank University, London SE1 0AA, U.K.

## Abstract

Mixed tin–lead (Sn–Pb) halide perovskites, with their tunable bandgaps (1.2–1.4 eV), show great promise for the development of highly efficient all-perovskite tandem solar cells. However, achieving commercial viability and stabilized high efficiency for Sn–Pb perovskite solar cells (PSCs) presents numerous challenges. Among various optimization strategies, the incorporation of additives has proven critical in modulating the crystallization of Sn–Pb perovskites. Despite the widespread use of additives to improve performance, detailed photophysical mechanisms remain unclear. In this work, we elucidate the mechanistic role of guanidinium thiocyanate, a chaotropic agent, in the crystallization of Sn–Pb perovskites. We combine hyperspectral imaging with real-time in situ photoluminescence spectroscopy to study the crystallization process of Sn–Pb perovskites. Our findings reveal that the chaotropic agent modulates the crystal growth rate during perovskite crystallization, resulting in more homogeneous films with reduced nonradiative recombination. We challenge the common assumption that crystallization stops once the solvent evaporates by identifying photoluminescence variations during the cooldown process. The resulting films exhibit a photoluminescence quantum yield of 7.28% and a charge carrier lifetime exceeding 11 μs, leading to a device efficiency of 22.34% and a fill factor of over 80%. This work provides a fundamental understanding of additive-mediated crystal growth and transient cooldown dynamics, advancing the design of high-quality Sn–Pb perovskites for efficient and stable optoelectronics.

## Graphical abstract

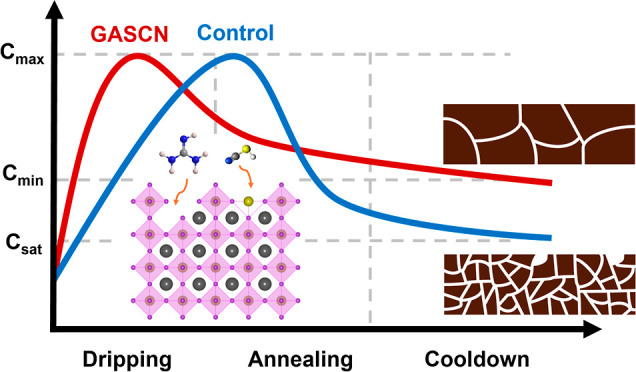

## Introduction

Organic–inorganic metal halide perovskites have attracted enormous attention throughout the optoelectronics research community due to their outstanding optoelectronic properties and wide applications. Perovskite solar cells (PSCs) using lead (Pb) perovskites can now exceed 26% power conversion efficiency (PCE), retaining over 92% of their certified PCE (25.2%) after 2500 h of operation.^1,2^ However, the energy bandgaps of Pb perovskites are larger than the ideal bandgap for the narrow-gap component of all-perovskite tandems.^3,4^ Mixed tin–lead (Sn–Pb) perovskites will therefore play a pivotal role in fabricating highly efficient all-perovskite tandems because their bandgap can be tuned to around 1.2 eV. Nevertheless, the journey toward commercial viability and long-term stability of Sn–Pb PSCs is fraught with challenges: the easy oxidation of Sn^2+^ to Sn^4+^ accelerates the degradation and affects device longevity;^5^ high defect densities of Sn–Pb perovskites increase charge recombination rates and lead to drops in performance.^6^ Among various strategies to optimize the properties of Sn–Pb perovskites, the incorporation of additives has been identified as a crucial approach for material engineering.^7,8^

Chaotropic agents based on urea or guanidine salts have been notably popular in improving the homogeneity of perovskite thin films.^9–12^ They disrupt the structure of secondary bonds such as Lewis acid–base interactions in the precursor solution,^13^ resulting in better device performance. Guanidinium thiocyanate (GASCN) stands out as a particularly influential chaotropic additive, for its role in improving the operational stability and efficiency of Pb-based PSCs.^14–19^ Some of the benefits include superior crystallinity,^16^ improved thermal stability,^20^ and reduced ion migration,^14^ all of which collectively enhance the performance and durability of the solar cells. In 2016, De Marco et al. demonstrated that guanidinium-based additives could significantly enhance carrier lifetimes and open-circuit voltages in hybrid PSCs, setting the stage for subsequent innovations.^21^ A study in 2017 showed that post-treating a MAPbI_3_ film with GASCN/IPA solution significantly improved the optoelectronic quality of the film.^14^ The resulting devices exhibited an order of magnitude improvement in charge carrier lifetime, leading to an enhanced PCE. A later study by the same group revealed that doping the perovskite with 2% GA effectively suppressed current–voltage hysteresis between forward and reverse scan in devices.^22^ The incorporation of GA cations into the MAPbI_3_ crystal structure was also reported by Jodlowski et al. to stabilize device performance for 1000 h under continuous light illumination, marking a fundamental achievement in the perovskite field.^23^ In addition to Pb perovskites, researchers explored the effect of GA cations on Sn perovskites. In 2018, Jokar et al. combined GA cations with FA in FASnI_3_ perovskite and found fewer pinholes in the resulting film.^24^ By incorporating 20% GA cations, they were able to increase the *V*_oc_ from 0.51 to 0.62 V. Furthermore, the charge carrier lifetime doubled from 0.7 to 1.4 ns.

In 2019, efficient large guanidinium Sn–Pb PSCs were reported to have enhanced photovoltage and low energy losses by Wu et al.^25^ Subsequently, Tong et al. incorporated GASCN as an additive in narrow bandgap Sn–Pb ((FASnI_3_)_0.6_(MAPbI_3_)_0.4_) PSCs, leading to notable improvements in their structural and optoelectronic properties.^26^ The defect densities of the perovskite films were reduced 10-fold, resulting in charge carrier lifetimes exceeding 1 μs. Recently, in 2023, Ren et al. employed guanidinium salts for interfacial passivation in inverted PSCs, highlighting their role in reducing charge extraction barriers and enhancing device stability.^27^ This progression underscores the ongoing development and increasing sophistication of GASCN applications in PSCs.

Although GASCN is widely used in a range of perovskite compositions to boost performance, the underlying photophysical mechanisms through which it modulates the crystallization in mixed Sn–Pb PSCs are yet to be understood. In this work, we report on the use of GASCN as a chaotropic agent to improve the film quality and device performance of mixed Sn–Pb perovskites. We discuss trends observed in steady-state and time-resolved photoluminescence (TRPL) spectroscopy and compare the photoluminescence quantum yields (PLQY) of the polycrystalline films. Moreover, we utilize X-ray diffraction, hyperspectral imaging, and in situ photoluminescence monitoring to elucidate the role of GASCN during the crystallization step of the Sn–Pb perovskite thin films. This comprehensive photophysical characterization highlights fundamental aspects of additive engineering in Sn–Pb PSCs and paves the way for the development of more efficient and stable photovoltaic systems. By investigating the photophysical processes enhanced by GASCN, we provide a blueprint for the rational design of Sn–Pb PSCs, which could have far-reaching implications for the future of tandem PSCs.

## Results and Discussion

We used a one-step antisolvent technique to deposit a narrow bandgap mixed Sn–Pb perovskite, Cs_0.025_FA_0.475_MA_0.5_Sn_0.5_Pb_0.5_I_2.925_Br_0.075_. We prepared the precursor solution by adding a stoichiometric blend of the chemicals (listed in the Supporting Information) in a solvent mixture of dimethylformamide and dimethyl sulfoxide (DMF/DMSO). To modify these perovskite films, we introduced GASCN at different molar ratios with respect to the perovskite molar concentration. The device structure consisted of glass/indium tin oxide (ITO)/poly­(3,4-ethylenedioxythiophene)–poly­(styrenesulfonate) (PEDOT:PSS)/perovskite/fullerene (C_60_)/bathocuproine (BCP)/silver (Ag), as shown in Figure 1a. The total device active area of the single-junction cell was 0.18 cm^2^, and the mask aperture area was 0.1 cm^2^. All measurements were made with unencapsulated devices and performed in a N_2_-filled glovebox at room temperature. Figure 1b presents the *J*–*V* curves of champion devices both with and without GASCN addition, with the detailed photovoltaic (PV) parameters listed in the figure.

**Figure 1:**
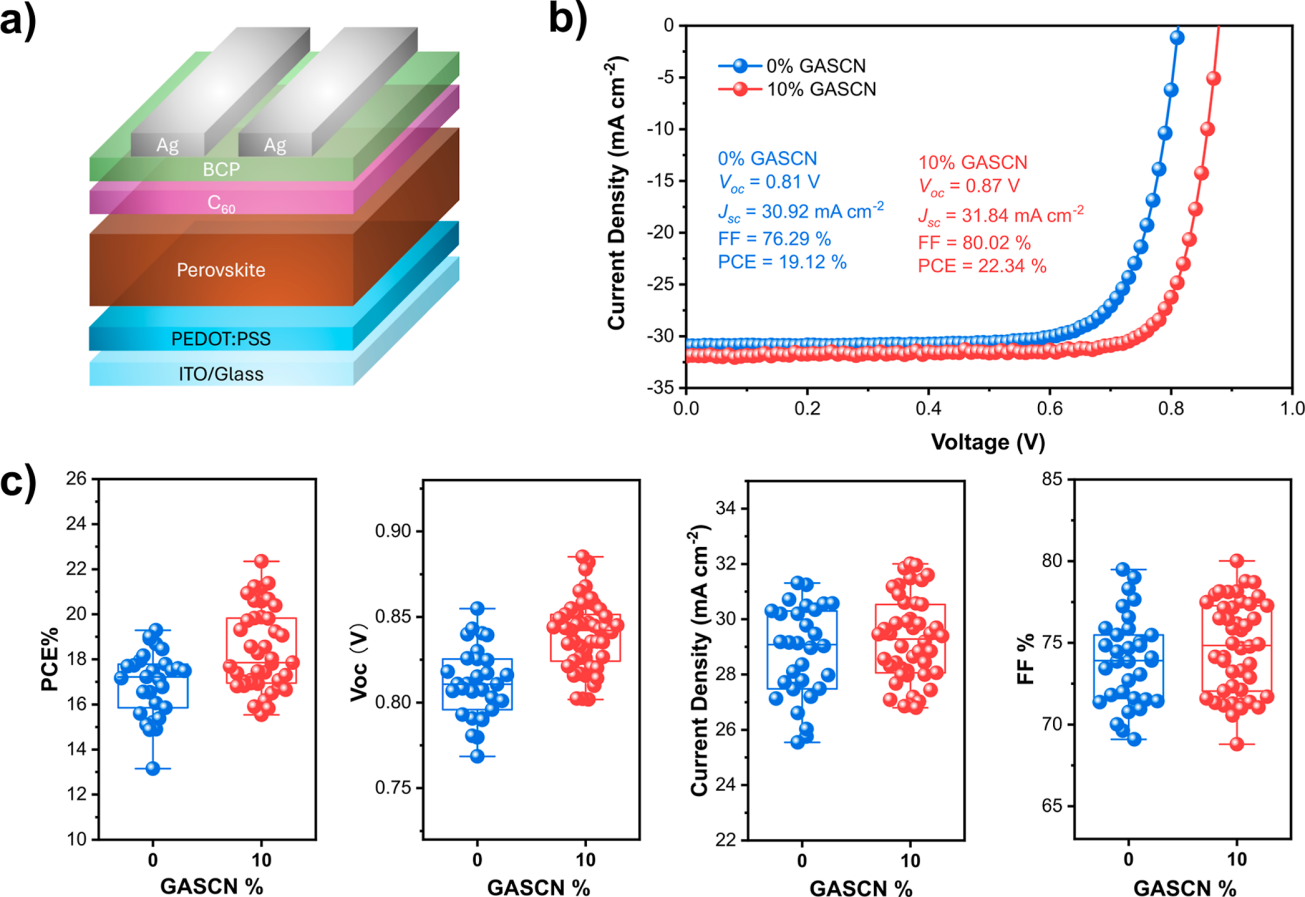
(a) Schematic of the p–i–n perovskite solar cell. (b) *J*–*V* characteristics of the champion PSCs without and with GASCN. (c) PV parameters as statistical distribution for ∼30 PSCs without and with GASCN.

The champion control devices achieved a PCE of 19.12%, *J*_sc_ of 30.92 mA cm^–2^, *V*_oc_ of 0.81 V, and an FF of 76.29%. In contrast, devices with 10% GASCN showed a PCE of 22.34%, a *J*_sc_ of 31.84 mA cm^–2^, a *V*_oc_ of 0.87 V, and an FF of 80.02%. In Figure S1, we show forward and reverse *J*–*V* scans for devices with and without GASCN addition, which both show negligible hysteresis. The external quantum efficiency (EQE) spectra of both devices are shown in Figure S2, with the integrated photocurrent density (31.13 mA cm^–2^) over the AM 1.5G solar spectrum aligning with the *J*_sc_ value from *J*–*V* characterization. Figure 1c illustrates the statistical distribution of all PV parameters for approximately 30 devices each, both 0% GASCN and 10% GASCN. Although the average PV parameters were enhanced for PSCs with 10% GASCN, the most significant improvement was observed in *V*_oc_, with minor enhancements in *J*_sc_ and FF. We also investigated the effect of varying GASCN concentrations (0, 5, 10, and 20%) on device characteristics. All PV parameters systematically improved as the GASCN concentration increased from 0% to 10% but declined when the concentration went further to 20% (Figure S3). Although stability was not the focal point of this study, Figure S4 shows the performance of the PSCs after both 624 and 984 h, both showing a decrease in PCE (Table S1). The reduced stability of Sn–Pb PSCs is often attributed to the facile oxidation of Sn^2+^ to Sn^4+^ upon air exposure, which, along with other chemical processes,^28–30^ can breakdown the perovskite phase and form recombination centers.^31,32^ Additionally, the presence of Sn^4+^ leads to unwanted p-doping, which can affect recombination dynamics and interfacial energetic alignment within the devices.^33–35^ However, we have recently demonstrated that the stability of Sn–Pb PSCs is mainly affected by PEDOT:PSS, and dedoping it can reduce PCE loss from 37% to under 7% after 1100 h in an inert atmosphere.^36^

To further confirm that the improvement in performance is unique to GASCN, we investigated the effects of other additives, specifically chaotropic agents guanidinium iodide (GAI) and sodium thiocyanate (NaSCN), on device performance. As shown in Figure S5 and Table S2, while GAI and NaSCN both influenced device behavior, neither demonstrated a consistent enhancement in PCE comparable to GASCN. The champion device treated with GASCN achieved the highest PCE of 22.34%, in contrast, GAI resulted in a significant decrease in performance, with a champion PCE of only 15.40%, while NaSCN showed no meaningful improvement, reaching a champion PCE of 18.13%, similar to the control (19.12%). These results confirm that the performance enhancement is specifically attributed to GASCN rather than the general effect of guanidinium- or thiocyanate-based additives.

To investigate why 10% GASCN was the most favorable additive amount, we first turn to scanning electron microscopy (SEM) to examine the surface morphology of the perovskite films. The SEM of Sn–Pb perovskite is characterized by clusters of crystal grains as shown in Figure 2a,b.^37^ Figure 2a,b displays a significant difference in surface morphology, with the 10% GASCN having significantly larger cluster size than the 0% film. Additionally, although an increase in cluster size with increasing GASCN is apparent, as shown in Figure S3, a more careful look at the SEM image for the 20% GASCN reveals fine particles emerging from the surface that may contribute to poor crystallization kinetics and increased grain boundaries. Thus, while moderate GASCN concentrations (10%) resulted in well-merged clusters with reduced grain boundary density, the 20% GASCN films exhibited a mix of large and irregularly shaped clusters with voids at grain boundaries. Previous studies have shown that this morphology disrupted charge transport pathways and increased nonradiative recombination sites,^26,38–40^ which could explain our observed decrease in PCE.

**Figure 2:**
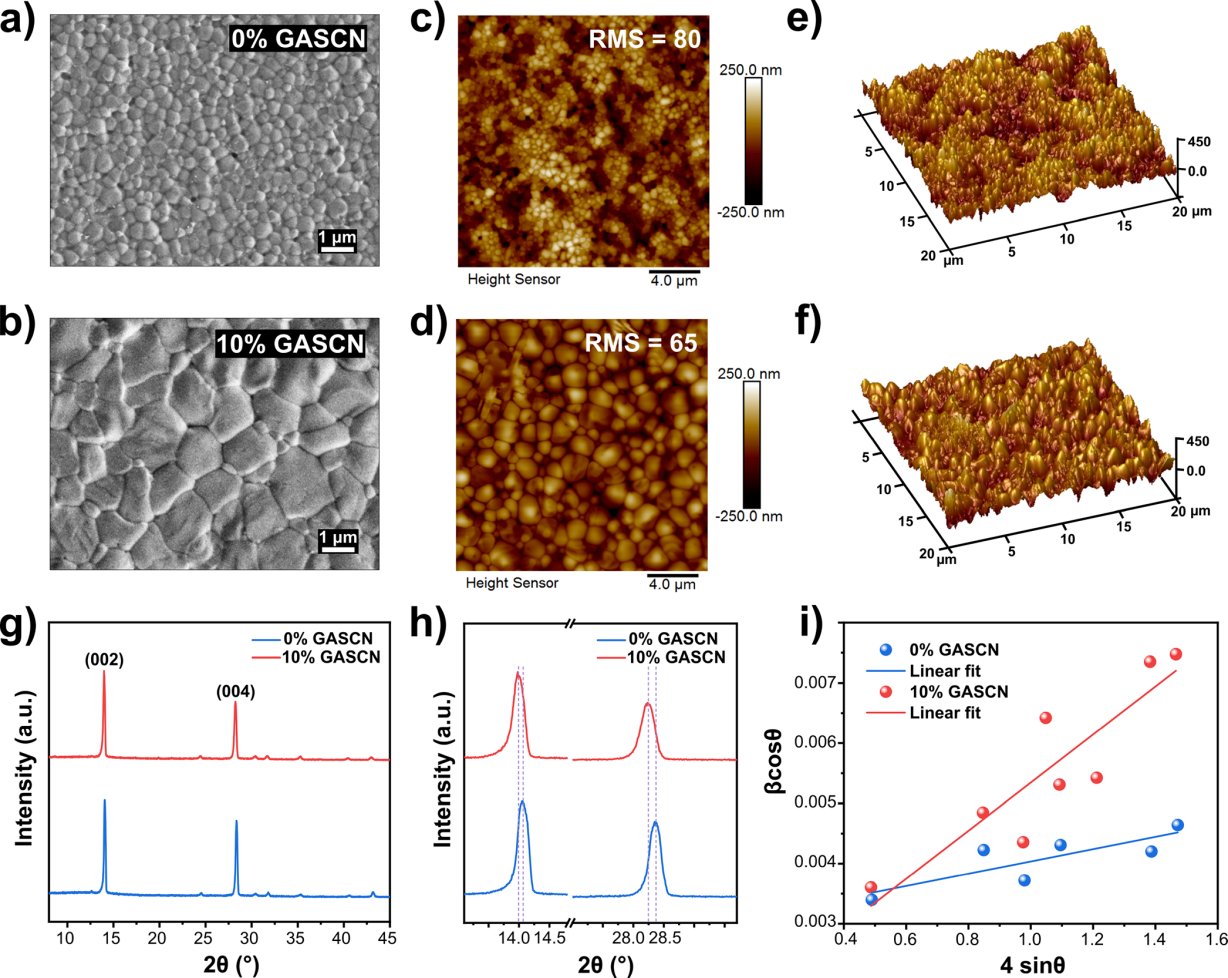
Top-view SEM images of (a) 0% GASCN and (b) 10% GASCN films. Top-view topographical AFM images of (c) 0% GASCN and (d) 10% GASCN films. 3D AFM images of (e) 0% GASCN and (f) 10% GASCN films. (g) XRD patterns of 0 and 10% GASCN films. (h) Magnification of the XRD peaks at (002) (left) and (004) (right) of 0 and 10% GASCN films. (i) Williamson–Hall plots used to determine microstrain and crystallite size for the Sn–Pb perovskite films with 0 and 10% of GASCN.

We also utilized atomic force microscopy (AFM) to examine the surface morphological alterations of perovskite thin films both without GASCN and with 10% GASCN, as illustrated in Figure 2c,d,e,f. The root-mean-square roughness (RMS) of the thin films decreased from 80 nm in the 0% GASCN film to 65 nm in the film containing 10% GASCN. The decrease in surface roughness likely led to improved charge carrier dynamics by minimizing trap sites, enhancing charge transport, and reducing recombination. This smoother surface also optimized interface contact for more efficient charge extraction,^41–44^ contributing to the higher device performance we discussed earlier. Consequently, we determine that 10% GASCN is the optimal amount for improving device performance and perovskite film quality; therefore, the following characterization focuses on the comparison between 0% and 10% GASCN films.

The X-ray diffraction (XRD) patterns in Figures 2g and S6 show all the films to match the expected cubic structure of Cs_0.025_FA_0.475_MA_0.5_Sn_0.5_Pb_0.5_I_2.925_Br_0.075_ perovskite; however, weak additional peaks matching to PbI_2_ (at 12.7°) and In_2_O_3_ (at 30.6° and 35.4°) of the ITO substrate are also visible.^45^ The peak for PbI_2_ reduced in intensity with increasing guanidinium cation incorporation, suggesting the use of the additive suppresses secondary phase formation as also observed by other reports.^46–48^ High intensities for the peaks centered at 14.1° and 28.4° indicated a strong preference for the (002) and (004) planes of the perovskite. The (002)/(004) peak intensity ratio however was similar across all samples, indicating that the GASCN additive did not participate in any preferential growth.^49^ As the GASCN concentration increased, the peaks shifted to lower angles, as shown in Figures 2h and S6. This indicated the incorporation of the larger guanidinium cation by substitution of the perovskite cation,^50^ resulting in a gradual expansion of the unit-cell volume.^23,51,52^ Although the GA cation was present in the unit cell, the addition of GASCN did not lead to the formation of a 1D Ga perovskite structure, as confirmed by the absence of XRD peaks near 8° and 11°.^23^

Williamson–Hall analysis (Figures 2i and S7 and Table S3) of the XRD data was carried out to quantify the microstrain caused by the GA cation to the unit cell.^53^ A residual microstrain value of 1.02 × 10^–3^ was observed for the pristine film, presumably due to the perovskite layer being grown on nonlattice matched substrates.^54^ For the 10% GASCN Sn–Pb perovskite film, an increase in microstrain to 3.99 × 10^–3^ was observed due to incorporation of the GA cation. Considering the increase in the calculated microstrain together with the observed shifting of the XRD Bragg peaks to lower 2θ values (Figures 2h and S6), this suggests the strain to be tensile over compressive due to GA incorporation. The relationship between the nature of the strain to device performance and stability is complicated, resulting in positive effects for some perovskite systems while being detrimental to others.^54–56^ In our case, an enhancement in performance was likely seen owing to a reduction of nonradiative recombination and voltage loss with the 10% GASCN additive. The Williamson–Hall results also suggested that the GASCN additive caused an increase in crystallite size, following the cluster size trend observed via SEM. A maximum crystallite size of >100 nm was observed for the 10% GASCN sample compared to <50 nm for the sample with 0% GASCN. This increase in crystallite size observed by XRD (and cluster size by SEM) was possibly caused by the constituent ions of GASCN stabilizing a mesophase and thus extending the time for crystallization of the perovskite film.^57^

To better understand the enhanced device performance, we examined the photophysical properties of the perovskite films. Figure S8a displays the ultraviolet–visible–near-infrared absorption spectra of the perovskite thin films. As shown in the spectra, the addition of 10% GASCN showed a negligible change in the bandgap. Figure 3a presents the steady-state photoluminescence (PL) characteristics of films with 0% and 10% GASCN. The PL peak intensity of the 10% GASCN film was approximately four times greater than that of the film without GASCN. The Urbach energy decreased from 16.32 meV in the 0% GASCN film to 15.54 meV with 10% GASCN, indicating reduced energetic disorder (Figures S9 and S10). These values were obtained from the PL spectral tail following the procedure in Ugur et al.^58^ Figure 3b shows the PL decays from TRPL measurements for 0% and 10% GASCN films. We fitted the decays in both cases to a stretched exponential (Figures S10 and S11), $PL\left(t\right)={A\cdot e}^{-{\left(t/\tau \right)}^{b}}$ , where *A* is a constant, *t* is the time after excitation, τ is the average lifetime, and *b* is a stretching factor that describes the amount of heterogeneity in the system.^59^ Using this analysis, we found that the 0% GASCN film had a lifetime of 3.08 μs, which was increased to 11.32 μs after treatment with 10% GASCN. We then measured the photoluminescence quantum yield (PLQY) as 3.40% for the 0% GASCN film and 7.28% for the 10% GASCN film (Figures 3c and S8d).

**Figure 3:**
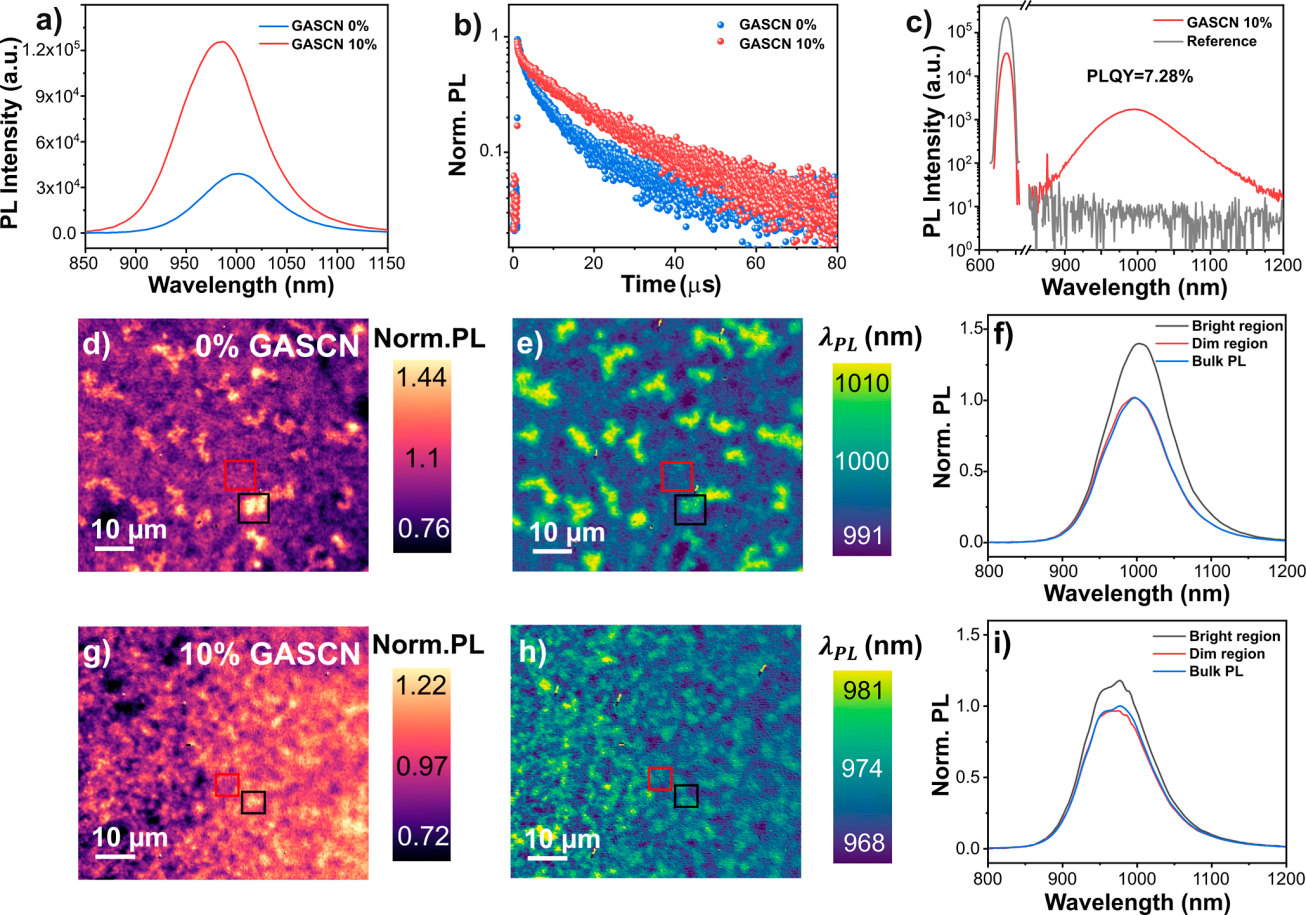
(a) Photoluminescence (PL) spectra of Sn–Pb perovskite films with 0% and 10% GASCN additive. (b) Time-resolved photoluminescence (TRPL) decays of Sn–Pb perovskite films with 0% and 10% GASCN additive. Excitation wavelength, 635 nm. Emission wavelength, 985 nm. (c) PL spectra of Sn–Pb perovskite films with 10% GASCN (red) and glass reference (gray) used to find the photoluminescence quantum yield (PLQY). (d) Normalized PL intensity map and (e) peak wavelength map of the 0% GASCN film. (f) Local normalized PL spectra taken in the labeled bright (black) and dim (red) regions of the 0% GASCN hyperspectral images in (d,e). (g) Normalized PL intensity maps and (h) peak wavelength map of the 10% GASCN film. (i) Local normalized PL spectra taken in the labeled bright (black) and dim (red) regions of the 10% GASCN hyperspectral images in (g,h). The scale bars in all images are 10 μm.

Taken together, the ensemble PL spectroscopy results helped build a picture of the role of GASCN in film photophysics. The concomitant increase in PL intensity and lifetime in moving from 0% to 10% GASCN indicated a lower nonradiative recombination rate in the latter. These changes in PL properties were likely due to the more controlled crystal growth in 10% GASCN-treated samples, which in turn should lead to lower trap densities.^21,60^ The addition of GASCN resulted in a blue shift of the PL peak, which could be attributed to a reduction in trap states within the perovskite^61^ and potential passivation interaction from the GASCN.^62^ Furthermore, the blue shift observed in the PL occurred without a significant change in the absorption edge (Figure S8a), while the Urbach energy decreased notably (Figures S9 and S10). These results suggested that GASCN effectively reduced band-tail states in the perovskite films.^63^ X-ray photoelectron spectroscopy (XPS) analysis further supported this interpretation, revealing a notable reduction in the relative concentration of Sn^4+^ species in the 10% GASCN films (Figure S13), consistent with a suppression of Sn oxidation and improved surface passivation.^64^ The resultant increase in PLQY from 3.40% to 7.28% implied, from the Ross relation,^65^ a decrease in nonradiative voltage loss, Δ*V*_nr_ from 87 to 68 meV, which accounted for some of the improvement in *V*_oc_ (∼60 mV) that we observed in devices. We posit that the additional improvements in *V*_oc_ may be due changes in band alignment at the interface between the perovskite and charge transport layers.^66^

Next, we performed hyperspectral PL microscopy on Sn–Pb perovskite films with 0% and 10% GASCN. Hyperspectral PL microscopy provides detailed insights into thin film semiconductors by enabling the simultaneous characterization of both spatial and spectral heterogeneities in photoluminescence.^67,68^ We provide details of the measurement in Supporting Information. Figure 3d–f shows normalized PL intensity and peak wavelength maps, as well as local spectra from “bright” and “dark” regions of a Sn–Pb perovskite film without GASCN treatment. We provide the same information in Figure 3g–i for a Sn–Pb perovskite film with the GASCN treatment.

The hyperspectral measurements (Figure 3d,g) show that the 10% GASCN-treated perovskite is ∼5 times more homogeneous in PL intensity (standard deviation, σ_PL_ = 0.0723) than the untreated perovskite (σ_PL_ = 0.171). The untreated perovskite was highly heterogeneous on the microscale, characterized by regions of blue-shifted, low-intensity and red-shifted, high-intensity PL. We posit that this type of heterogeneity, in which the local energy varies, is due to local trap states present in the 0% GASCN film.^69^ Overall, these hyperspectral PL images suggested that adding 10% GASCN resulted in more uniform and higher-quality perovskite films.

To further explore the origin of the high PL emission intensity with GASCN addition, we employed in situ PL to observe the crystallization process of the perovskite in real time. Unlike other characterization methods, the in situ PL technique directly evidences the evolution of the PL during the formation of the perovskite, making it a powerful method for examining the crystallization dynamics related to additive engineering as they occur.^70^ The experimental setup for the in situ PL measurements is shown in Figure 4a. PL emissions were recorded for approximately 55 s during the spin-coating (antisolvent-dripping at ∼20 s) stage and 240 s during the annealing stage (anneal at 100 °C), which was then followed by a 240 s cooldown period between 20 and 25 °C.

**Figure 4:**
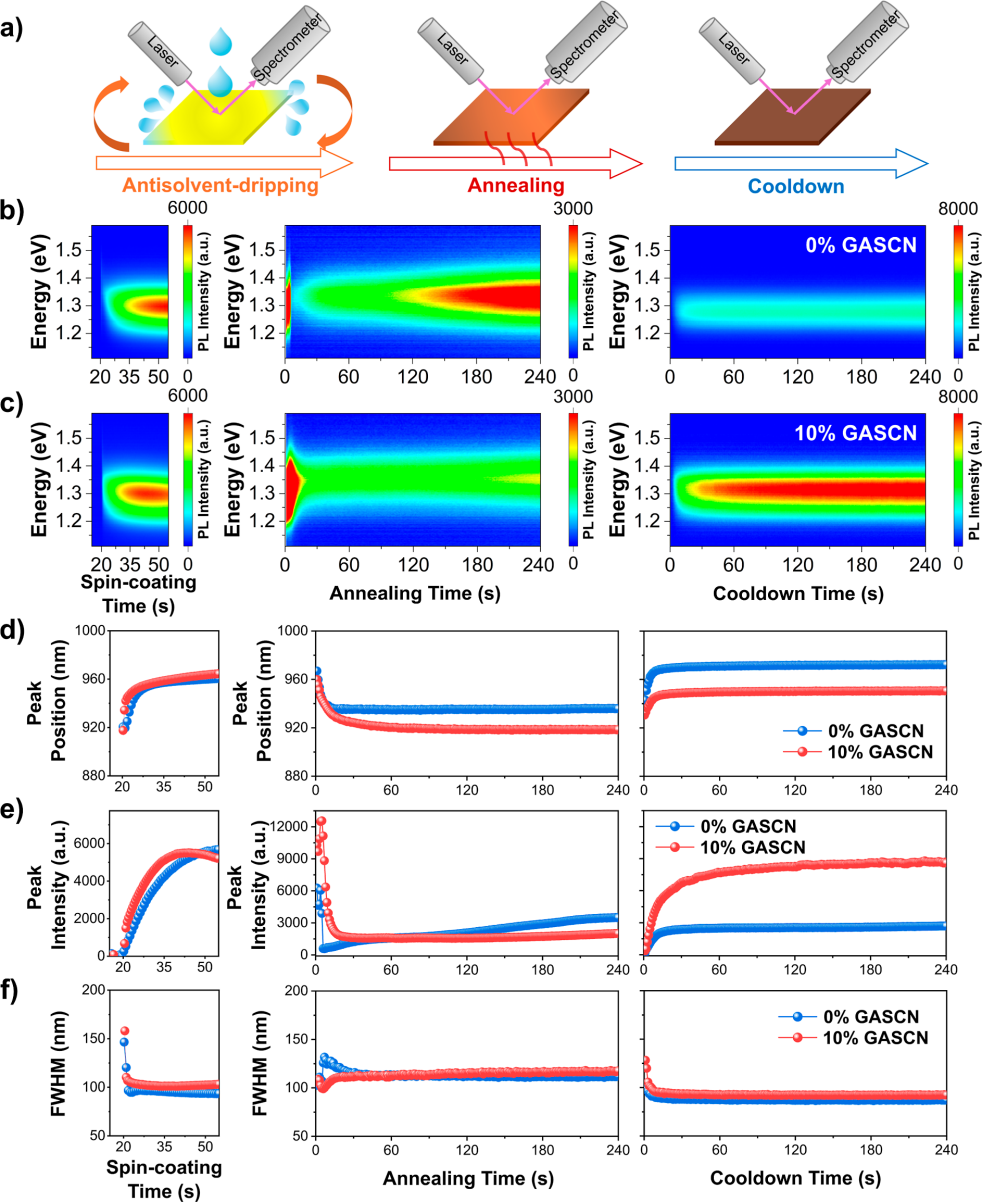
In situ PL measurements of the formation process of Sn–Pb perovskite films. Excitation wavelength = 405 nm. (a) Illustration of the in situ PL setup during antisolvent-dripping, annealing, and cooldown processes of perovskite film formation. The contour plots of the PL spectra during antisolvent-dripping (left), annealing (middle), and cooldown (right) stage of (b) Sn–Pb perovskite films without the GASCN additive and (c) Sn–Pb perovskite films with the 10% GASCN additive. Peak information plots obtained from in situ PL measurements for (d) peak position, (e) peak intensity, and (f) peak FWHM. Antisolvent dripping started at ∼20 s following spin-coating.

We present the PL spectra contour plots during the formation of Sn–Pb perovskite films with 0% and 10% GASCN in Figure 4b,c, while Figure 4d–f illustrates the extracted peak position, intensity, and full width at half-maximum (FWHM) using Gaussian fits. Both films exhibited initial PL signals at higher energy at around 1.39 eV (Figure 4d) compared to the final bulk emission energy at around 1.24 eV that we saw previously in Figure 3. Importantly, the light exposure during measurements did not induce the formation of additional perovskite phases, such as 2D perovskites (around 2 eV), as evidenced by the consistent spectral features shown in Figures S14 and S15. This observation confirmed that the structural integrity of the perovskite phase remained intact under the experimental conditions used.^71^

PL intensity changes can be associated with nucleation and crystal growth processes. The data in Figure 4d show that intensity peaked during spin-coating at 55 and 42 s for the 0% and 10% GASCN films, respectively. There was a red shift of the emission peak which was also faster for 10% GASCN and attributed to quantum-confined nanometer-scale nucleation and subsequent crystal growth,^72–74^ as well as compositional evolution from ion exchange, where higher-bandgap species formed initially.^75–77^ The earlier PL intensity peaks at 42 s for the 10% GASCN film compared to 55 s for the 0% GASCN film during spin-coating suggested that GASCN reduces the energy barrier for nucleus formation,^78^ potentially promoting spontaneous and more homogeneous nucleation across the film.

The emission peak position transition during annealing showed slower overall changes, which we attribute to slower crystal growth for the 10% GASCN film (Figure 4d). There were more significant but still slower changes in PL intensity for the 10% GASCN film during annealing (Figure 4e, middle), with continued slower PL intensity growth for 10% GASCN as the film cooled down (Figure 4e, right); both of these effects were consistent with slower growth. During the cooldown period following thermal annealing, both samples exhibited an increase in PL intensity, which can largely be attributed to the suppression of thermally activated nonradiative recombination as temperature decreased—a behavior consistent with typical semiconductor thermal quenching recovery.^79,80^ However, in the 10% GASCN sample, the increase in the PL intensity was notably smoother and more gradual, extending beyond what was expected from thermal effects alone. This distinct trend suggested that crystallization may still be ongoing during the cooling phase, further improving the structural order and enhancing radiative recombination. The irreversible nature of this PL evolution, unlike the behavior observed upon reheating and recooling (Figures S24 and S25), supported the interpretation that structural refinement continued even after the nominal annealing step. Figure 4f shows that the FWHM decreased upon spin-coating and stabilized during annealing and cooldown for both films, with the 10% GASCN film stabilizing faster compared to the 0% GASCN film. Both films displayed PL spectra that could be modeled by a single Gaussian.

Overall, the earlier nucleation triggered by antisolvent dripping, combined with slower crystal growth during annealing and cooling, suggested that GASCN addition elongated the crystallization process. This prolonged growth window allowed for more gradual and complete ripening and the integration of GASCN, leading to the formation of larger and higher-quality crystals, which was consistent with our observations from SEM and XRD.

The SCN^–^ ion, acting as a Lewis base, likely moderated the crystallization kinetics, promoting better structural organization and enabling the gradual enlargement of crystals,^81–83^ while the bulky guanidinium cation further influenced this process by slowing crystal growth, potentially enhancing defect passivation and film uniformity.^84^ During and after annealing, GASCN significantly impacted the crystallization process, allowing for more controlled growth and possibly better defect passivation (as referenced in the PL discussion). This was reflected in the sustained increase in PL intensity during the cooling stage for the 10% GASCN film, in contrast to the stabilization observed in the 0% GASCN film. Interestingly, the transient response of the PL during the cooldown process revealed significant changes that challenge the common assumption that crystallization stops once the solvent dries on the hot plate. This study demonstrated that substantial variations continued during the cooldown phase, which may play a decisive role in determining the final film properties. The increase in the PL intensity observed may largely result from changes occurring during the cooldown phase, highlighting its significance in the overall process. This finding provides valuable insight, delivering a clear message about the critical role of cooldown dynamics.

In order to explore the effects of other chaotropic agents on Sn–Pb film crystallization dynamics, we also explored GAI and NaSCN as additives. While these additives influenced crystallization behavior (Figures S16–S21), neither induced the simultaneous enhancement of the nucleation rate and prolonged crystal growth altogether as observed with GASCN. Regarding the cooldown dynamics, we observed that the PL peak intensity of NaSCN-containing films increased upon cooling, similar to GASCN. However, this increase occurred much more rapidly, suggesting that, unlike GASCN, NaSCN does not facilitate continued crystal growth after cooldown,^79,80,85^ with minimal contribution from passivation or postcooling structural evolution. These behaviors aligned well with the lower device performance observed in NaSCN and GAI films, indicating that the absence of sustained crystal growth and passivation effects may have contributed to increased defect densities and, therefore, suboptimal optoelectronic properties. Our finding suggest that the effects observed for GASCN may be exclusive to the combined presence of guanidium cations and thiocyanate anions rather than a general consequence of any chaotropic agents, highlighting the importance of their synergistic interaction in controlling perovskite crystallization and postcooling evolution.^70^

To further investigate the thermal stability of the films, we conducted additional measurements by reheating the films to 75 °C after deposition and monitoring their response upon subsequent cooling (Figures S22–S25). The results indicated that the PL enhancement was partially reversible at elevated temperatures (Figure S24), with GASCN films demonstrating superior resistance to thermal stress compared with other chaotropic agents. This was evidenced by their stable peak position, higher retained PL, and minimal peak broadening (Figures S23 and S25), suggesting that GASCN helped mitigate thermal degradation, possibly by stabilizing the perovskite lattice and reducing defect activation. These findings highlighted the complex interplay among crystallization dynamics, defect passivation, and thermal stability, underscoring the need for further studies incorporating long-term illumination stability tests to fully evaluate the implications of these effects under operational conditions.

To better illustrate the differences in crystallization pathways between control and GASCN-treated Sn–Pb perovskite films, we constructed Figure 5 as a mechanistic schematic based on our experimental observations and prior discussion. As depicted in Figure 5a, the addition of 10% GASCN leads to rapid supersaturation during the antisolvent dripping stage, crossing the nucleation threshold earlier and triggering a denser nucleation event compared to the control. While this would typically be expected to result in smaller grains, our morphological (SEM, AFM) and structural (XRD) data instead showed significantly larger and more uniform clusters in GASCN-containing films. This apparent contradiction was reconciled by invoking Ostwald ripening during annealing, where small, high-energy crystallites redissolved and contributed to the growth of larger domains. Supporting this, in situ PL data revealed a substantial drop in PL intensity following slow intensity increase during the annealing stage for 10% GASCN films—likely indicative of the transient dissolution or reorganization of smaller crystals (Figure 4c). In contrast, the control film exhibited no such slow PL intensity change during annealing (Figure 4b), suggesting minimal Ostwald ripening and limited postnucleation reorganization. This was consistent with the smaller, more fragmented grains observed in control samples, as well as their inferior optoelectronic properties.

**Figure 5:**
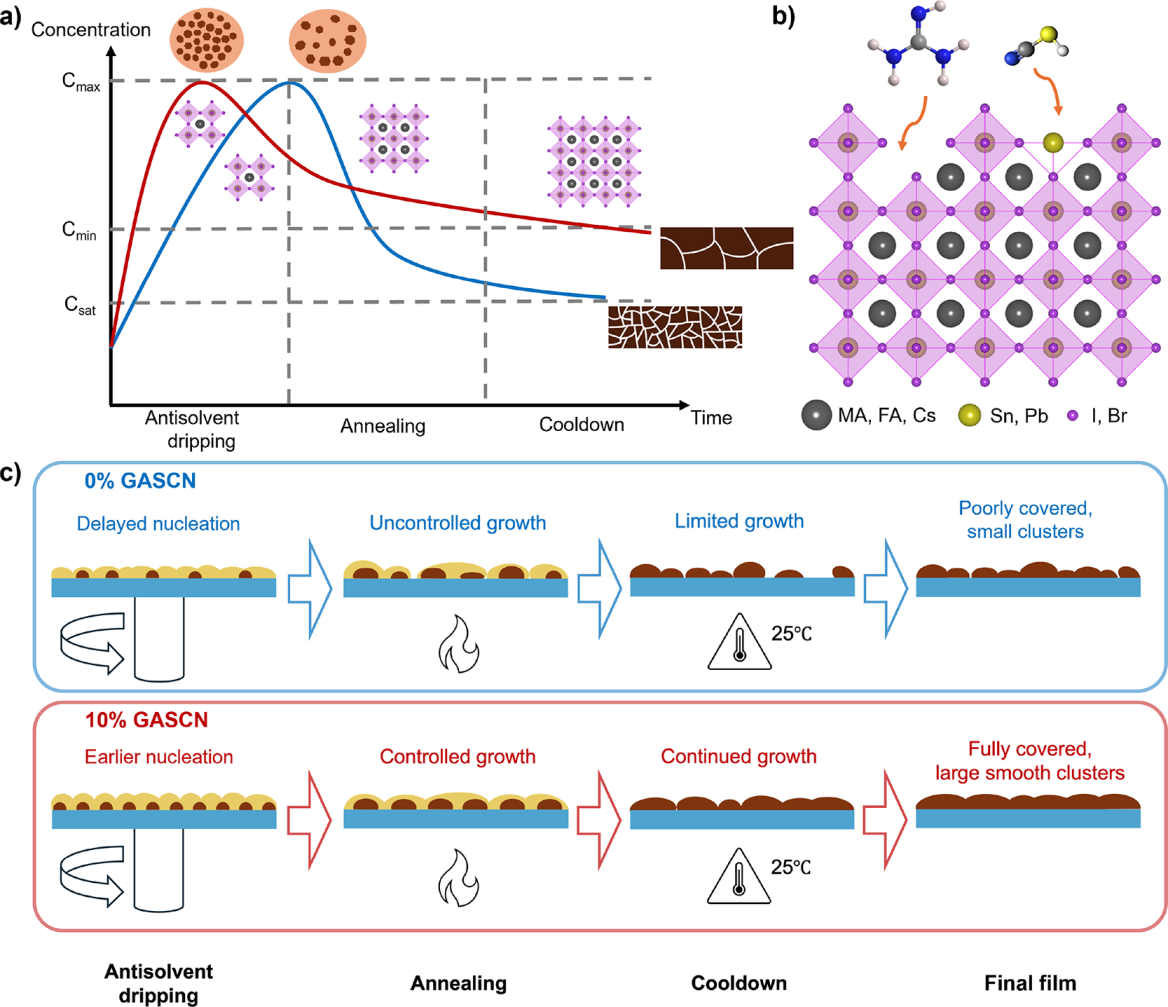
Mechanistic illustration of the crystallization pathway in Sn–Pb perovskite films with and without GASCN. (a) Conceptual La Mer-type concentration–time profile showing the differences in nucleation and growth dynamics for control (0% GASCN, blue curve) and 10% GASCN (red curve) films across three key stages: antisolvent dripping, annealing, and cooldown. (b) Schematic representation of guanidinium (GA^+^) and thiocyanate (SCN^–^) ions interacting with the Sn–Pb perovskite lattice, potentially contributing to defect passivation. (c) Stepwise comparison of film evolution for control (0% GASCN, top, blue panel) and GASCN-containing (bottom, red panel) conditions.

The benefits of GASCN treatment extend beyond the annealing stage. As Figure 5c illustrates, continued growth during the cooldown phase contributes to the formation of smooth, well-connected grain clusters. This prolonged crystallization behavior, further confirmed by in situ PL results (Figure 4e), challenges the conventional assumption that crystallization stops immediately after solvent evaporation. Figure 5b highlights a possible chemical basis for this effect: both guanidinium cations and thiocyanate anions may interact with the perovskite lattice to passivate defects such as undercoordinated A-sites or halide vacancies. This passivation not only reduces nonradiative recombination (as reflected in the improved PLQY and TRPL decay dynamics, Figure 3b,c) but may also stabilize intermediates and slow down crystallization kinetics, enabling a more controlled, energetically favorable grain evolution.

In short, the mechanistic illustration presented in Figure 5 captures the essence of how chaotropic agent GASCN enables superior film formation through a sequence of early nucleation, enhanced Ostwald ripening, and extended cooldown growth, mechanisms that are largely suppressed in the control film. This proposed model integrates all experimental insights and highlights the critical role of additive engineering in modulating crystallization pathways for high-performance Sn–Pb PSCs.

In summary, the results presented in this study offer a promising pathway for the future development of high-efficiency Sn–Pb PSCs. By employing GASCN as a chaotropic agent, we achieved a significant modulation of crystal growth, leading to improved film quality and optoelectronic properties. To further improve the long-term operational durability of Sn–Pb PSCs, replacing acidic and hygroscopic PEDOT:PSS with alternative hole transport layers (HTLs) or employing a HTL-free architecture will be essential.^36,86^ Such modifications would address the stability challenges posed by current HTLs (as discussed above), enabling the development of more robust devices.

Moreover, our findings suggest that the strategic use of chaotropic agents in Sn–Pb halide perovskites could pave the way for new design rules, particularly by enabling more controlled crystal growth in this family of narrow bandgap perovskites. We anticipate that other common chaotropic agents, such as urea and its derivatives, imidazolium salts, hydroxylamine compounds, and sulfonic acid–base agents, could also hold significant promise. Continued efforts to develop novel chaotropic agents and optimize their integration into perovskite layers will drive the development of new materials with enhanced stability and performance for optoelectronic applications.

## Conclusion

In this work, we demonstrated that the chaotropic agent GASCN played a crucial role in modulating crystal growth in Sn–Pb halide perovskites. Its incorporation resulted in increased grain size, reduced surface roughness, and enhanced photophysical properties, including suppressed nonradiative recombination and prolonged charge carrier lifetimes. Real-time in situ PL measurements revealed that GASCN slowed the crystal growth rate and promoted the formation of homogeneous and high-quality perovskite films. Notably, the transient increase in PL intensity during the cooldown process underscored the critical importance of this often-overlooked stage in determining the final optoelectronic properties of the films. PSCs incorporating GASCN achieved an impressive efficiency of 22.34%, underscoring its transformative impact on both crystallization and device performance. These findings established a clear framework for utilizing chaotropic agents to optimize narrow bandgap perovskites, setting the stage for the development of next-generation high-performance tandem solar cells.

## Supplementary Material



## Notes

Experimental details; forward and reverse *J*–*V* curves of 0% and 10% GASCN; EQE spectra of 0% and 10% GASCN devices; *J*–*V* curves of the champion with different amounts of GASCN; PV parameters as statistical distribution for devices with 0, 5, 10, and 20% GASCN; top-view SEM images of perovskite thin films made with 0, 5, 10, and 20% GASCN; *J*–*V* curves of 10% GASCN device after 624 and 984 h; detailed *J*–*V* parameters table; champion *J*–*V* curves for control (0% GASCN), 10% GAI, 10% GASCN, and 10% NaSCN device; device statistics table for control, 10% GAI, 10% GASCN, and 10% NaSCN devices; XRD patterns of 0, 5, 10, and 20% GASCN films; Williamson–Hall plots for the Sb–Pb perovskite films with varying amounts of GASCN; Williamson–Hall results’ table; UV–vis, photoluminescence (PL), and TRPL spectra and PLQY plot of Sn–Pb films with 0, 5, 10, and 20% GASCN; Urbach energy plot for 0% and 10% GASCN; TRPL and stretched fit for 0% and 10% GASCN films; XPS analysis of Sn 3d core levels in 0% and 10% GASCN films; contour plot of PL spectra for 0% and 10% GASCN films, ranging from 1.2 to 2.5 eV; contour plots of PL spectra for control (0% GASCN, without additive), 10% GAI, 10% GASCN, and 10% NaSCN films during spin-coating, annealing, and cooldown; peak information plots obtained from in situ PL measurements for peak position, peak intensity, and peak FWHM during spin-coating, annealing, and cooldown; contour plots of PL spectra for control, GAI, GASCN, and NaSCN films upon reheating at 75 °C and cooldown to 25 °C; and peak information plots obtained from in situ PL measurements for peak position, peak intensity, and peak FWHM upon reheating at 75 °C and cooldown to 25 °C (PDF)
