# Supplementary material for: Crystal Growth Modulation of Tin–Lead Halide Perovskites via Chaotropic Agent
Source: J Am Chem Soc. 2025 Aug 23;147(35):31578–90. doi: 10.1021/jacs.5c05772 (PMC12412113; doi:10.1021/jacs.5c05772)
Supplement: Supplementary file 1 [file ja5c05772_si_001.pdf]

Supporting Information

Crystal Growth Modulation of Tin-Lead Halide Perovskites via Chaotropic Agent

Yueyao Dong[1], Wen-Xian Zhu[2], Dong-Tai Wu[2], Xuan Li[4], Robert J. E. Westbrook[5], Chi-Jing Huang[2], Zeyin Min[4], Weiying Hong[4], Boyuan Wang[1], Ganghong Min[1], Sanjayan Sathasivam[6], Matteo Palma[4], Stoichko Dimitrov[4], Chieh-Ting Lin[2,3*], Thomas J. Macdonald[1*]

[1]*Department of Electronic & Electrical Engineering, Roberts Building, University College London, London WC1E 7JE, United Kingdom*

[2]*Department of Chemical Engineering, National Chung Hsing University, Taichung 40227, Taiwan*

[3]*Innovation and Development Center of Sustainable Agriculture, National Chung Hsing University, Taichung 40227, Taiwan*

[4]*Department of Chemistry, Queen Mary University of London, London E1 4NS, United Kingdom*

[5]*Department of Chemistry, University of Washington, Seattle, WA, 98195., United States of America*

[6]*School of Engineering, London South Bank University, London SE1 0AA, United Kingdom*

E-mail addresses: c.lin15@nchu.edu.tw (C-T. Lin), tom.macdonald@ucl.ac.uk (T.J. Macdonald).

Materials

Formamidinium iodide (FAI, >99.99%), Methylammonium iodide (MAI, >99.99%), Methylammonium bromide (MABr, >99.99%), Ethane-1,2-diammonium iodide ($EDAI_2$) was purchased from Great Solar Ltd. Tin(II) iodide (SnI_2, 99.999 metal trace), tin(II) fluoride (SnF_2, 99%), caesium iodide (CsI, 99.999%), glycine hydrochloride (GlyHCl, >99%), guanidinium thiocyanate (GASCN, >99%), guanidinium iodide (GAI, >99%), sodium thiocyanate (NaSCN, >99%), N,N-dimethylformamide (DMF, anhydrous 99.8%), dimethyl sulfoxide (DMSO, anhydrous 99.8%), Toluene (TOL, anhydrous 99.8%), 2-propanol (IPA, anhydrous 99.8%) was purchased from Sigma-Aldrich. Lead powder (99% metal trace) and fullerene (C_{60}, 99%) was purchased from alfa Aesar. Lead(II) iodide (PbI_2, 99.99% trace metal basis), lead(II) bromide ($PbBr_2$, >98%) was purchased from Tokyo Chemical Industry Co., Ltd. Bathocuproine (BCP, 99%) was purchased from Luminescence technology corp. Poly(3,4-ethylenedioxythiophene) polystyrenesulfonate (PEDOT:PSS, Clevios PVP Al 4083) was purchased from Heraeus Co., Ltd.

Preparation of Perovskite Precursor Solution

The preparation of a precursor solution for mixed tin-lead perovskite is divided into two parts. First, a 1.3M $Cs_{0.05}FA_{0.95}SnI_3$ solution was prepared by dissolving appropriate amount of CsI, FAI, SnI_2, SnF_2, and lead powder (10 mg) in mixed solvent of DMF:DMSO = 4:1. In the second part, a 1.3M $MAPbI_{2.85}Br_{0.15}$ solution was prepared by dissolving appropriate amount of MABr, $PbBr_2$, MAI, PbI_2, and GlyHCl (11.6 mg) in mixed solvent of DMF:DMSO = 9:1. Finally, Sn-Pb solution ($Cs_{0.025}FA_{0.475}MA_{0.5}Sn_{0.5}Pb_{0.5}I_{2.925}Br_{0.075}$) was obtained by mixing equal volumes of $Cs_{0.05}FA_{0.95}SnI_3$ and $MAPbI_{2.85}Br_{0.15}$. Different ratios of GASCN (5, 10, 20 %) relative to perovskite molar concentration are added to the Sn-Pb solution. For other chaotropic agents, 10% of GAI or NaSCN are added to the Sn-Pb solution.

Perovskite Thin Film and Device Fabrication

Glass/ITO substrates (15 Ω sq^{-1}, ruilong Ltd., Taiwan) were cleaned by sequentially sonication with deionized water, acetone, and IPA for 15 min each, before being dried under a stream on N_2 and treated with oxygen plasma for 10 min. PEDOT:PSS was fabricated from an aqueous dispersion which was filtered through a 0.45μm PVDF filter and then spin-coated at 4000 rpm for 20s on ITO substrate followed by heating at 150°C for 15min. The ITO/PEDOT:PSS film was then transferred to an N_2-filled glove box for the rest of the fabrication process. The

perovskite precursor was spun at 1000 rpm for 10 s followed by 5000 rpm for 50 s. 750μL toluene was dropped at 20th second from the beginning of the spin coating. The sample was then heated at 100°C for 10 min, followed by annealing at 65°C for 50 min. For the perovskite surface passivation, $EDAI_2$ solution (1 mg $EDAI_2$ was added to 1 mL IPA and 1 mL TOL) was spun at 4000 rpm for 20s followed by heating at 65°C for 10 min. Finally, C_{60} (40 nm), BCP (5 nm), and Ag (100 nm) were thermally deposited under high vacuum conditions (< 10^{-6} Pa) to complete the PSC fabrication process.

The total device active area of single-junction cell was 0.18 cm^2, and the mask aperture area was 0.1 cm^2. All measurements were made with unencapsulated devices and performed in a N_2-filled glove box at room temperature.

Characterizations

The current density-voltage (J-V) characteristics were measured under AM 1.5G solar simulator (SS-X, Enlitech). The light intensity of the illumination source was calibrated by a silicon reference cell. The solar cell device was measured with a 10 mV voltage step and a 20 ms time step using a Keithley 2400 source meter. Scanning electron microscope (SEM) images were obtained using the German brand Zeiss ULTRA PLUS under the accelerating voltage of 3 kV. Atomic force microscopy (AFM) images were obtained by employing a dimension icon scanning probe microscope (Bruker) and imaging in Peak Force Tapping™ mode via the use of Bruker ScanAsyst Air tips. UV-vis absorption measurements in the wavelength range from 600nm to 1100nm were conducted using a Shimadzu UV-1400 spectrometer. X-ray diffraction measurements were performed under D8 advance (Bruker) with a Cu Kα source. XPS measurements were performed on a Thermo Scientific Kα photoelectron spectrometer using Al Kα radiation. High resolution scans were acquired for the primary peaks of Pb 4f, Sn 3d and C 1s with a pass energy of 50 eV. Peak fitting was carried out using CasaXPS software and binding energies were charge corrected using adventitious carbon at 285 eV.

Photoluminescence Spectroscopy (ss-PL, TRPL, PLQY)

Steady state PL (ss-PL) spectra, time-resolved PL (TRPL) decay, and photoluminescence quantum yield (PLQY) were acquired using an Edinburgh Instruments FLS1000 spectrometer. For ss-PL spectra and PLQY measurements, a 450 W ozone-free Xenon arc lamp was used as excitation source and the detector was a liquid nitrogen-cooled near-Infrared photon multiplier tube (NIR-PMT) with spectral resolution from 600-1400 nm. TRPL decays were taken with an

Edinburgh Instruments HPLED 635nm picosecond pulsed diode laser. The repetition rate was controlled by an external trigger input and set to 12.5 kHz. The emission signal frequency was set to 3% that of the start rate to maintain single photon counting statistics. A NIR PMT-1400 detector was used for TRPL measurements. For PLQY measurements, the films were placed inside an integrating sphere (diameter = 150 mm), and the PLQY was calculated as:

$$PLQY = \frac{\int I_{ex}^{0} - \int I_{ex}^{s}}{\int I_{ems}^{s} - \int I_{ems}^{0}}$$

Where $\int I_{ex}^{0}$ and $\int I_{ex}^{s}$ represent the integrated intensities of the scattering peak for the reference and sample scans respectively, and $\int I_{ems}^{s}$ and $\int I_{ems}^{0}$ represent the integrated intensities of the emission peak for the reference and sample scans respectively. All samples were measured with glass encapsulation in a N_2 glovebox.

In situ Photoluminescence Spectroscopy

In situ PL spectroscopy was measured using a custom setup platform consisting of an Avantes HPLED 405nm laser at an intensity of 3.4 mW cm^{-2} and a Wasatch Photonics spectrometer coupled with optical fibers. Integration time of each spectrum was 500 ms during the measurement. All samples were measured under inert conditions in an N_2 glovebox.

Hyperspectral PL Microscopy

Hyperspectral measurements were performed using a Photon etc. Zephir 1.7x deep-cooled InGaAs sCMOS coupled to an IMA hyperspectral microscope with a 20X objective. The excitation was achieved with a 532 nm continuous wave laser, passing through a 532 nm laser line filter and emission was collected through a 500 nm dichroic filter and 550 nm long-pass filter. We specify the power used for individual measurements in the figure captions. The Hyperspectral Microscope uses a volume Bragg tunable filter to image a sample at specific wavelengths. The sample is imaged throughout the spectral range and these images are combined into a single four-dimensional database, or "cube", which carries the PL spectrum at each pixel. The pixel size was 108 x 108 nm and spatial resolution was diffraction limited. Images in the main text were line-by-line flattened in Python for clarity. All samples were

measured in inert conditions via glass encapsulation in a N_2 glovebox.

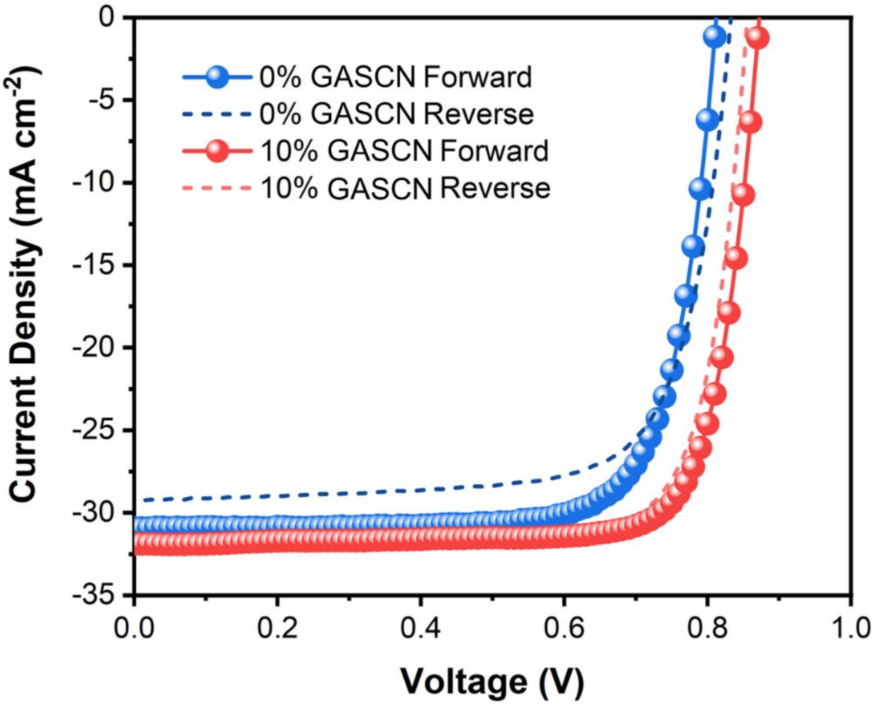

Figure S1. Forward and reverse *J-V* curves of 0% and 10% GASCN.

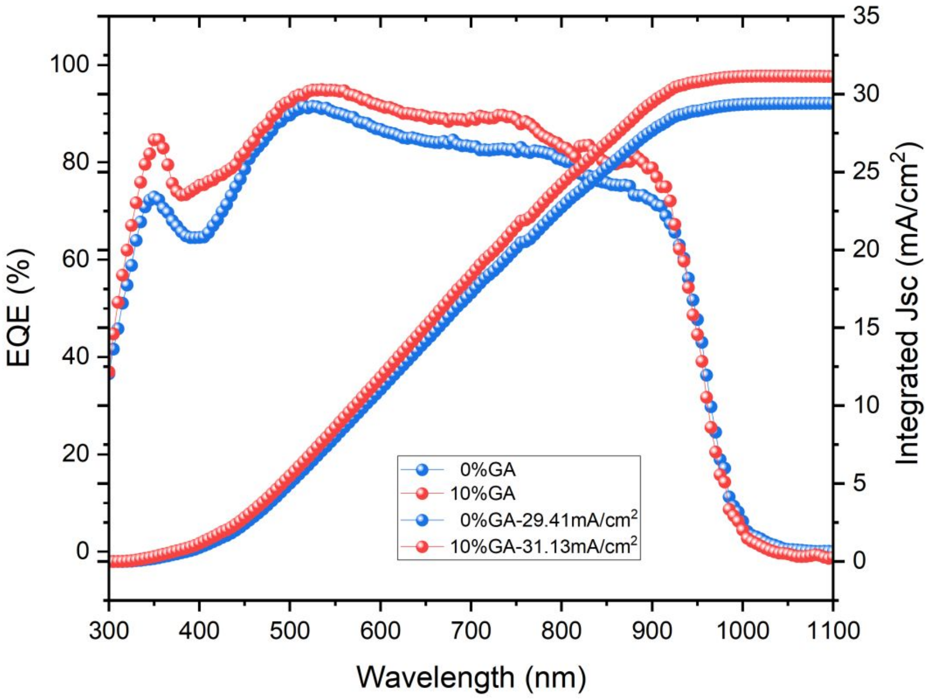

Figure S2. EQE spectra of 0% and 10% GASCN devices.

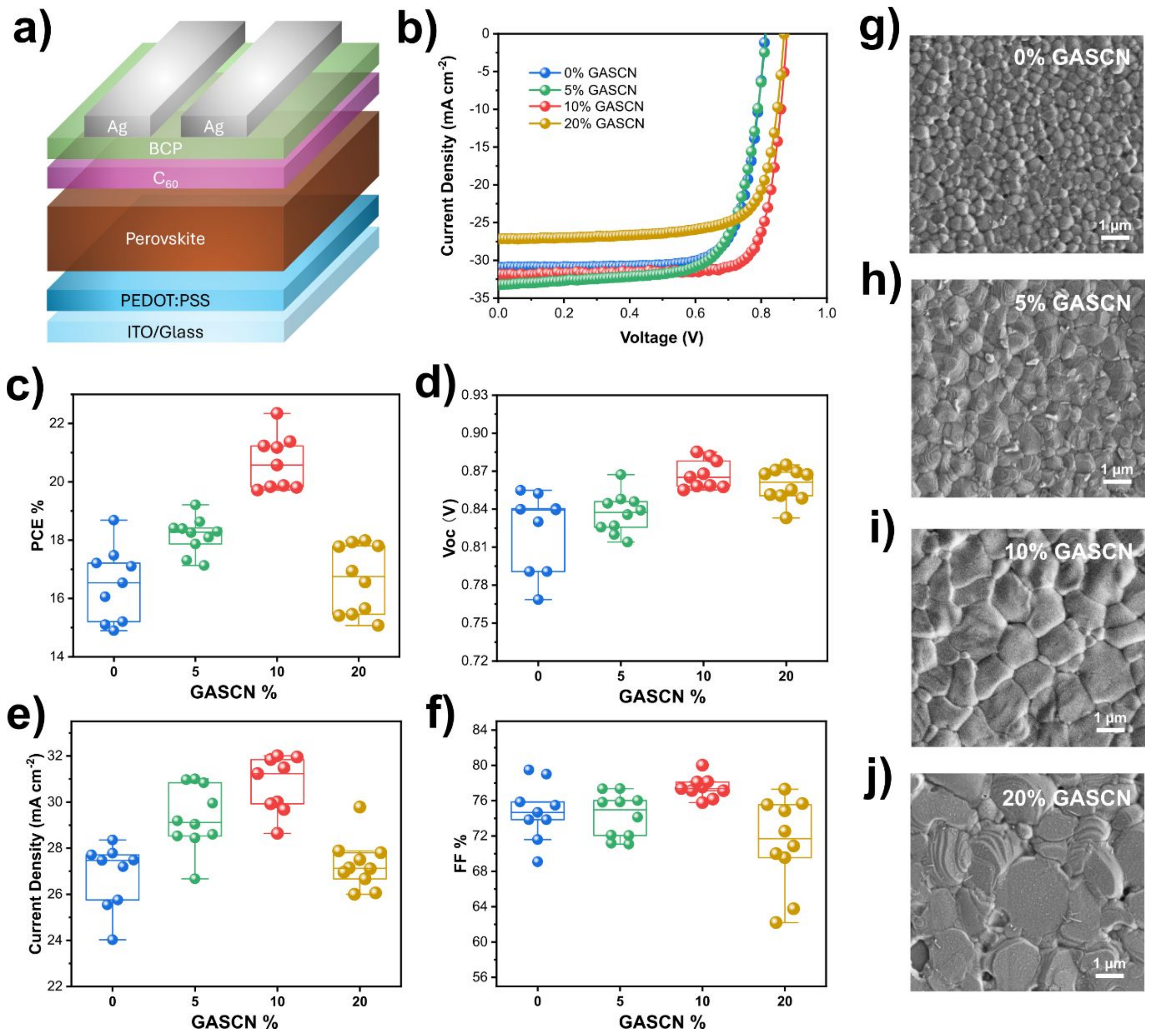

Figure S3. a) Schematic of the p-i-n perovskite solar cell. **b)** *J-V* characteristics of the champion with different amount of GASCN. **c)-f)** PV parameters as statistical distribution for devices with 0, 5, 10 and 20% GASCN. **g)-j)** Top-view SEM images of perovskite thin films made with 0, 5, 10 and 20% GASCN.

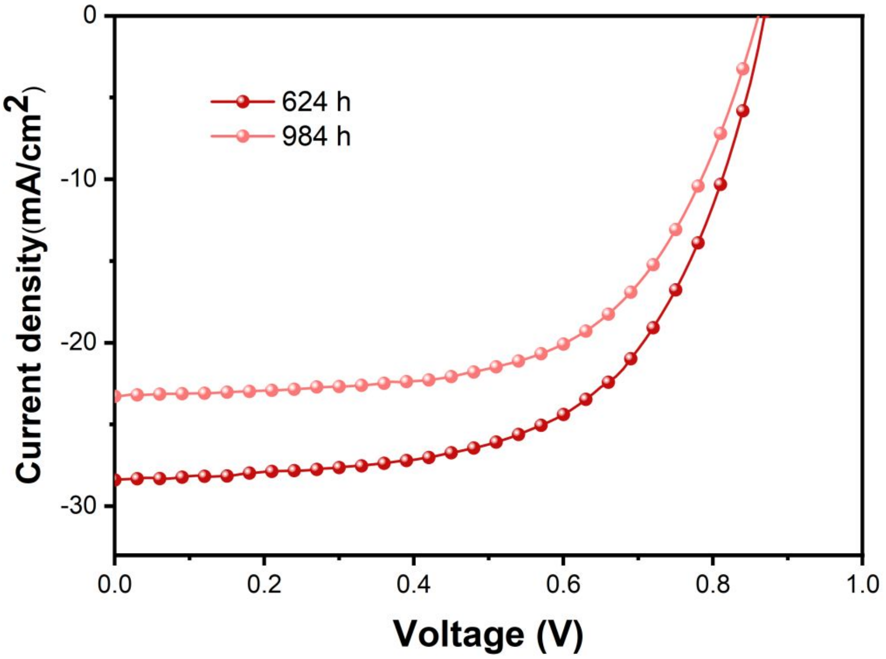

Figure S4. *J-V* curves of 10% GASCN device after 624 h and 984 h.

Time	V_{oc}	J_{sc}	FF	PCE
624 h	0.86856	28.38585	59.98971	14.79182
984 h	0.86055	23.20331	60.80927	12.12029

Table S1. Detailed *J-V* parameters from Figure S4.

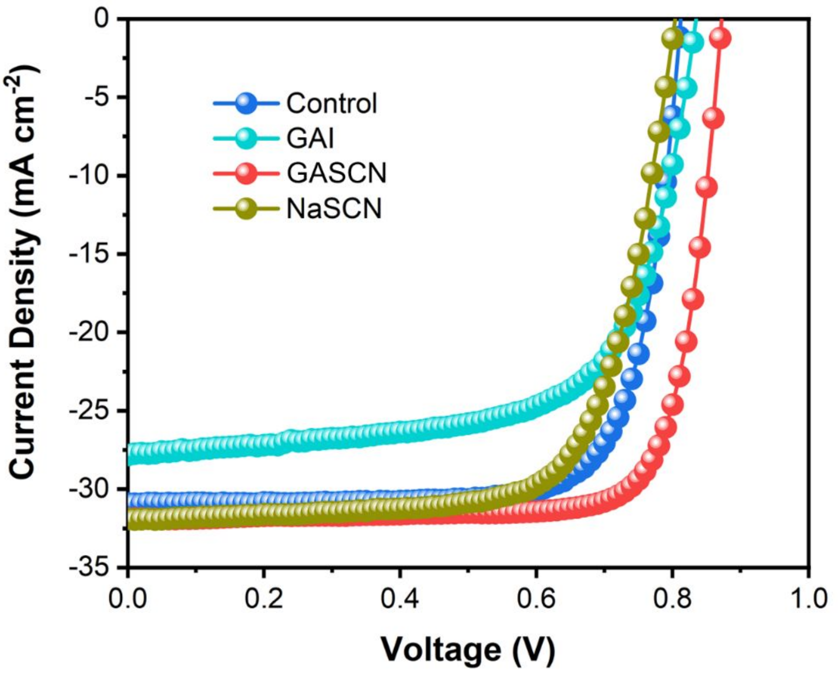

Figure S5. Champion *J-V* curves for control (0% GASCN), 10% GAI, 10% GASCN and 10% NaSCN device.

	PCE (%)	*Voc* (V)	*Jsc* (mA/cm^2)	*FF* (%)
**Control (0% GASCN)**	17.18±1.59 (19.12)	0.81±0.02 (0.81)	28.79±1.66 (30.92)	73.94±2.71 (76.29)
**GAI**	11.16±2.51 (15.40)	0.83±0.01 (0.83)	20.97±3.86 (27.79)	63.98±3.39 (66.48)
**GASCN**	18.40±1.80 (22.34)	0.83±0.02 (0.87)	29.40±1.76 (31.84)	74.72±2.83 (80.02)
**NaSCN**	17.24±0.85 (18.13)	0.78±0.02 (0.78)	30.90±1.32 (31.36)	71.06±0.78 (70.56)

Table S2. Device statistics for control, 10% GAI, 10% GASCN and 10% NaSCN devices.

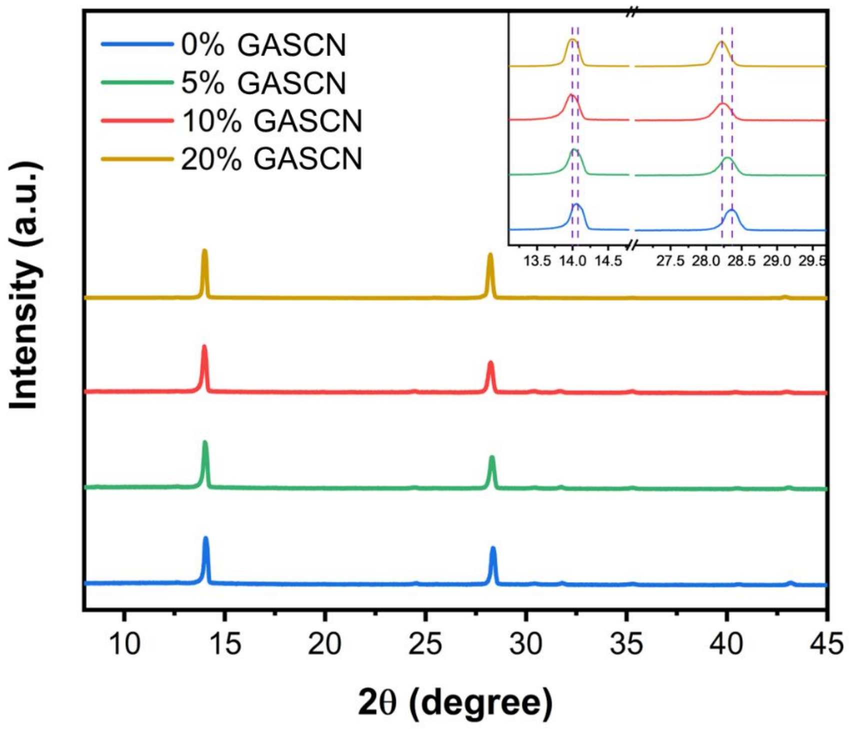

Figure S6. XRD patterns of 0, 5, 10, and 20% GASCN films. The low intensity peak at 12.7^o belongs to PbI_2 whereas the peaks at 30.6^o and 35.4^o match In_2O_3 from the ITO substrates.

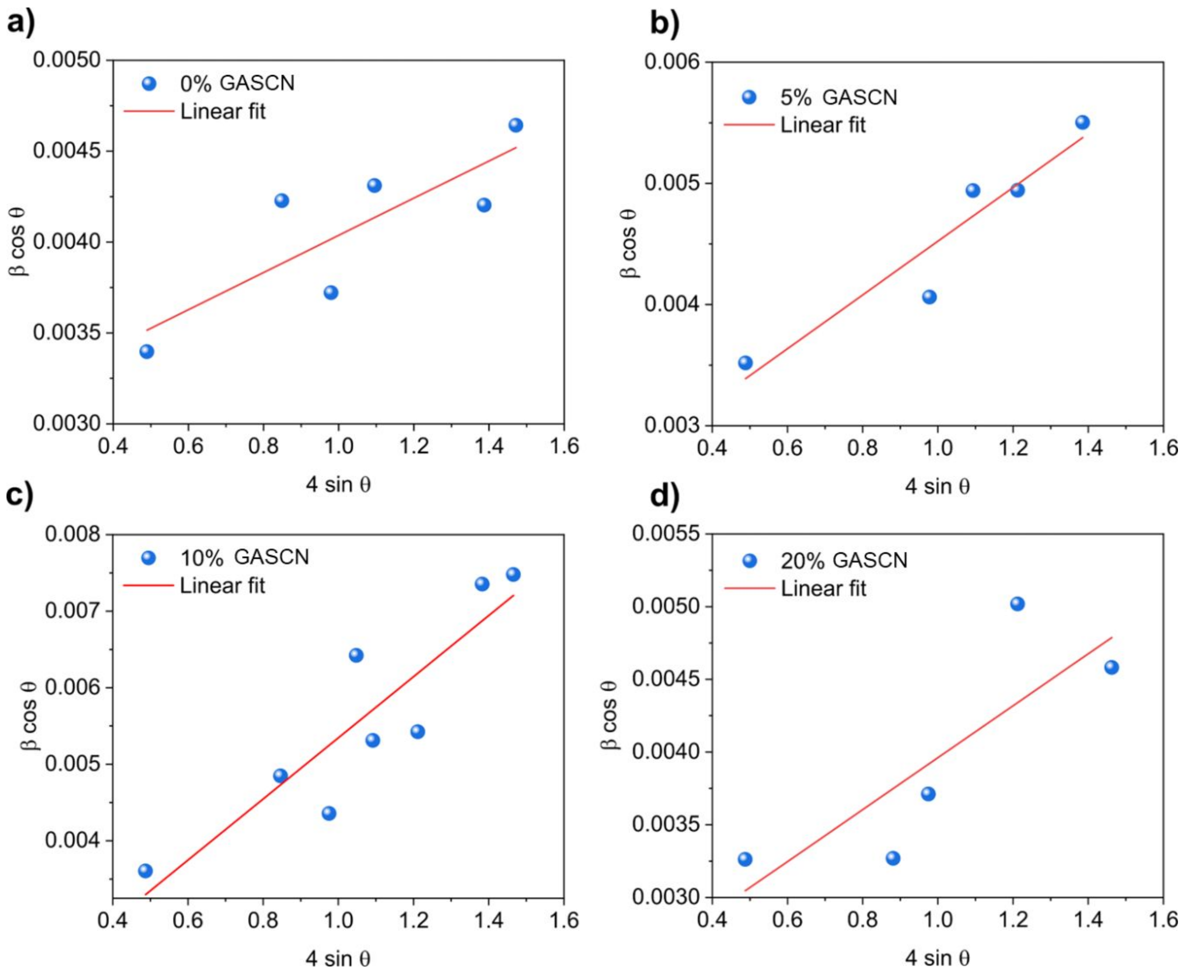

Figure S7. Williamson-Hall plots used to determine microstrain and crystallite size for the Sb-Pb films with varying amounts of GASCN.

Sample	Crystallite Size (nm)	Microstrain (x 10^{-3})
0	48	1.02
5	63	2.22
10	107	3.99
20	66	1.79

Table S3. Williamson-Hall results showing the change in crystallite size and microstrain due to GASCN additive.

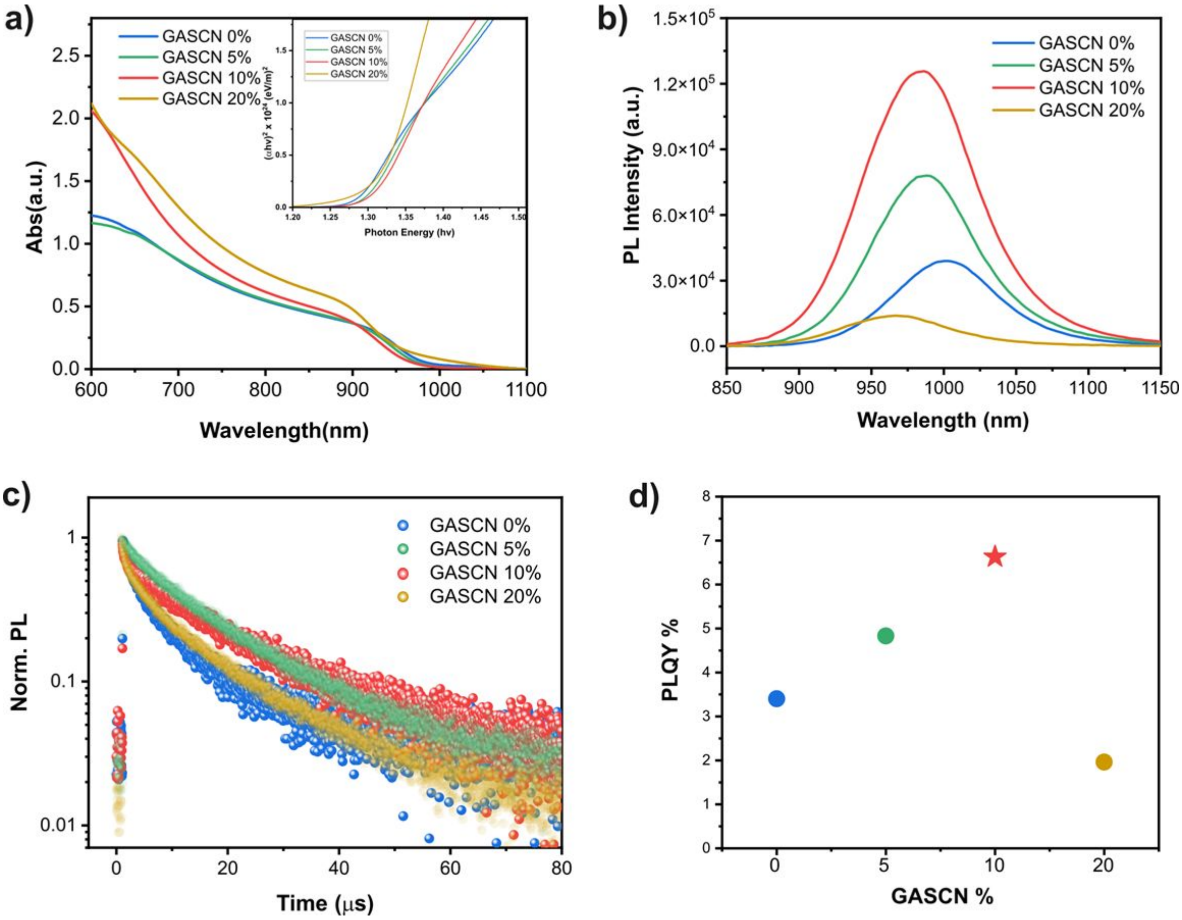

Figure S8. a) UV-Vis spectra of Sn-Pb films with 0, 5, 10, and 20% GASCN. **b)** Photoluminescence (PL) spectra of Sn-Pb films with 0, 5, 10, and 20% GASCN. **b)** Time-resolved photoluminescence (TRPL) spectra of Sn-Pb films with 0, 5, 10, and 20% GASCN. **d)** PLQY of Sn-Pb films with 0, 5, 10, and 20% GASCN.

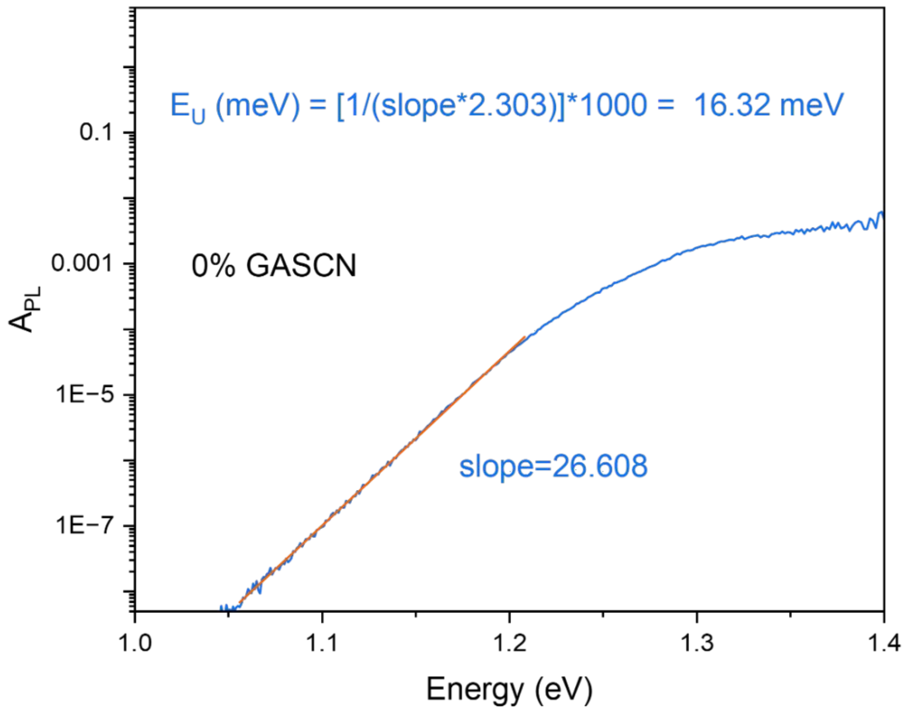

Figure S9. Urbach energy plot for 0% GASCN film.

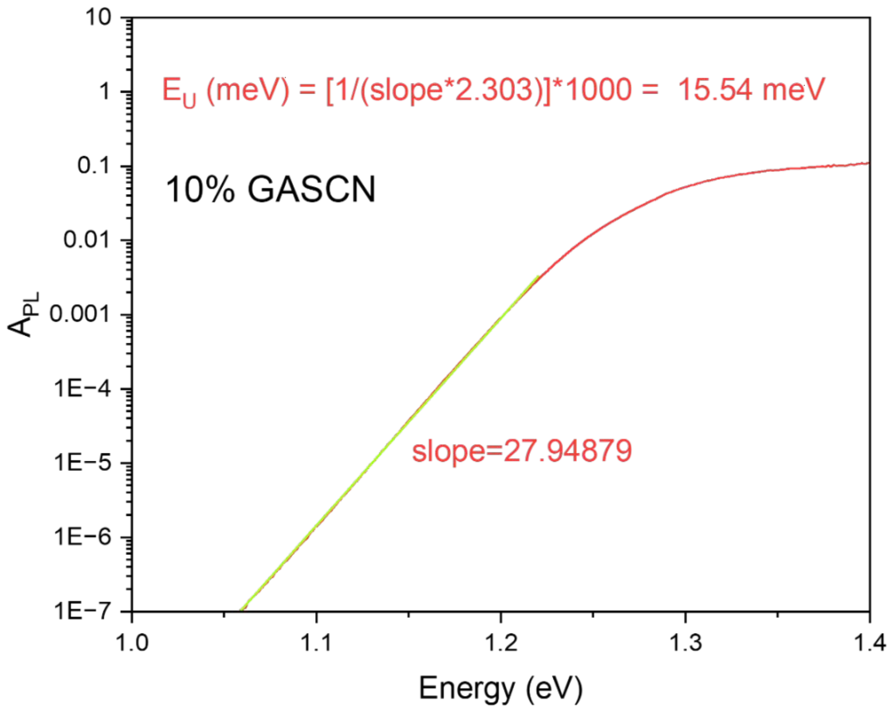

Figure S10. Urbach energy plot for 10% GASCN film.

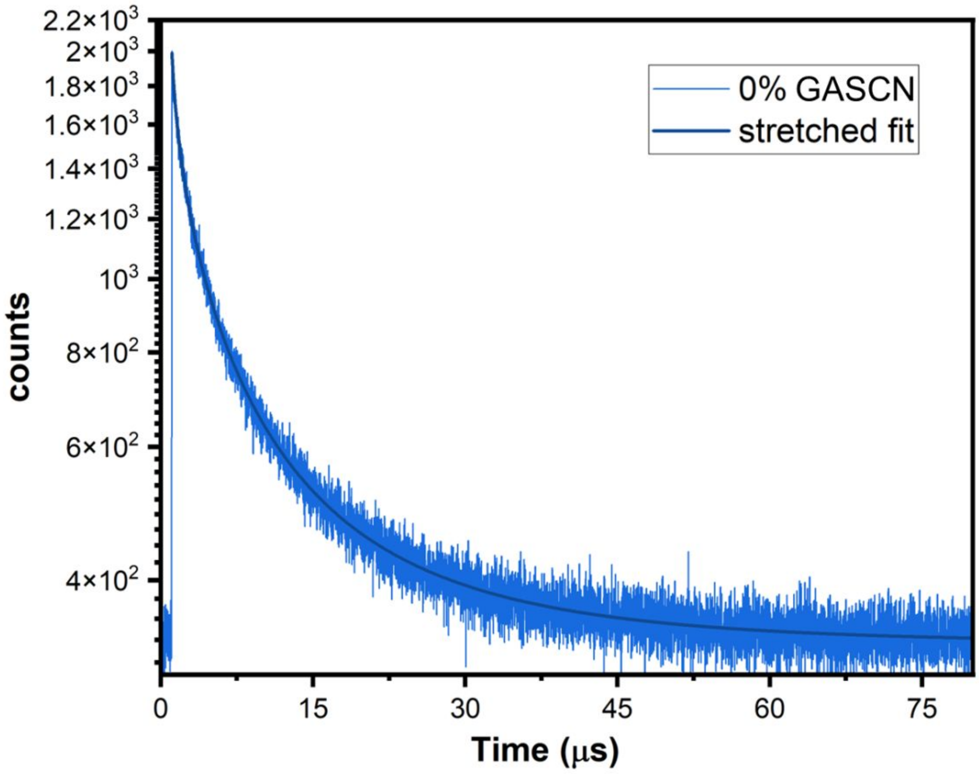

Figure S11. TRPL and stretched fit for 0% GASCN film.

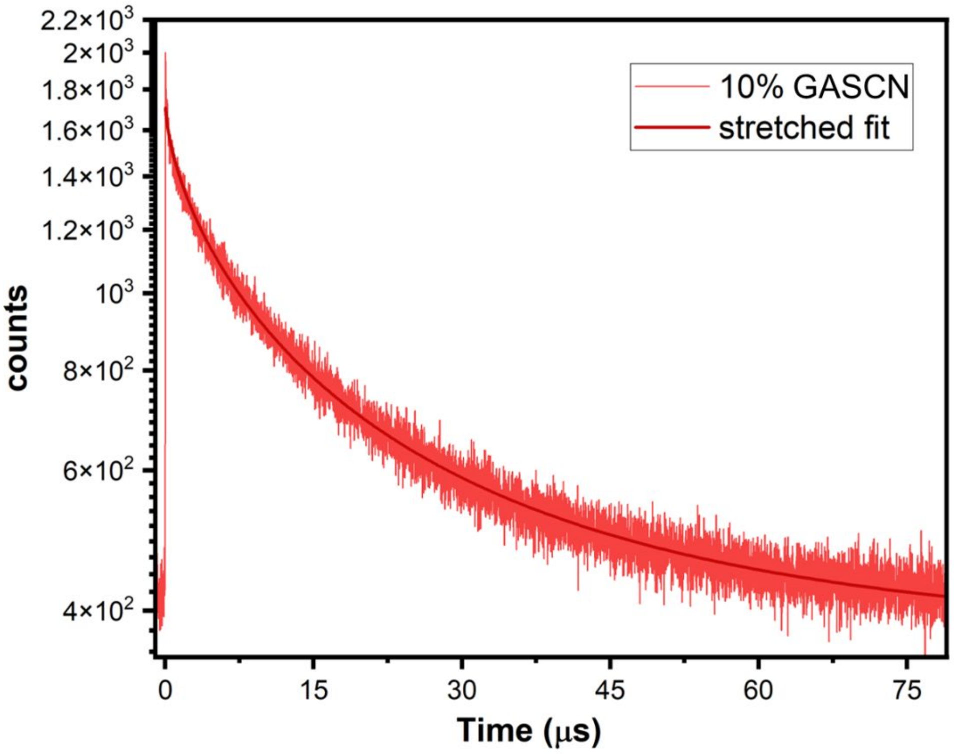

Figure S12. TRPL and stretched fit for 10% GASCN film.

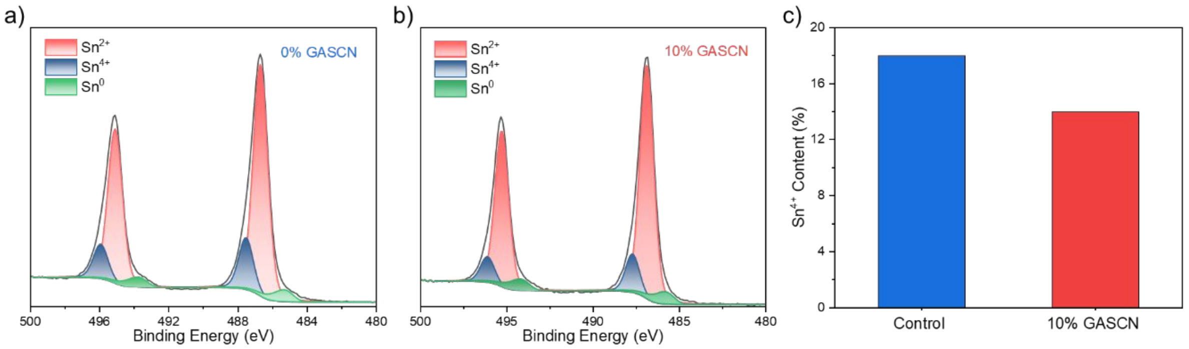

Figure S13. X-ray photoelectron spectroscopy (XPS) analysis of Sn 3d core levels in perovskite films. **a)** Sn 3d spectra of the control film (0% GASCN) and **b)** 10% GASCN film, showing deconvolution into Sn^0 (green), Sn^{2+} (red), and Sn^{4+} (blue) species. **c)** Quantification of Sn^{4+} content indicates a reduced level of Sn^{4+} in the GASCN-treated film.

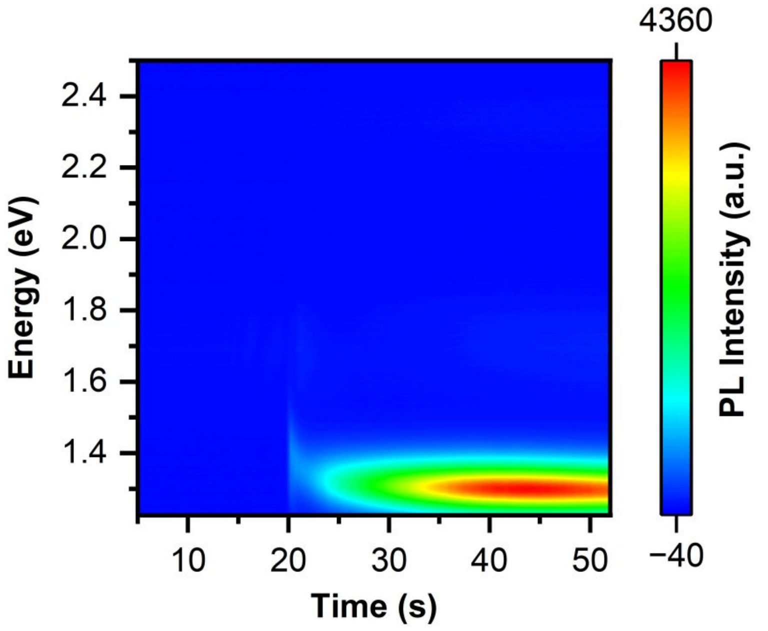

Figure S14. Contour plot of PL spectra for 0% GASCN film, ranging from 1.2 to 2.5 eV.

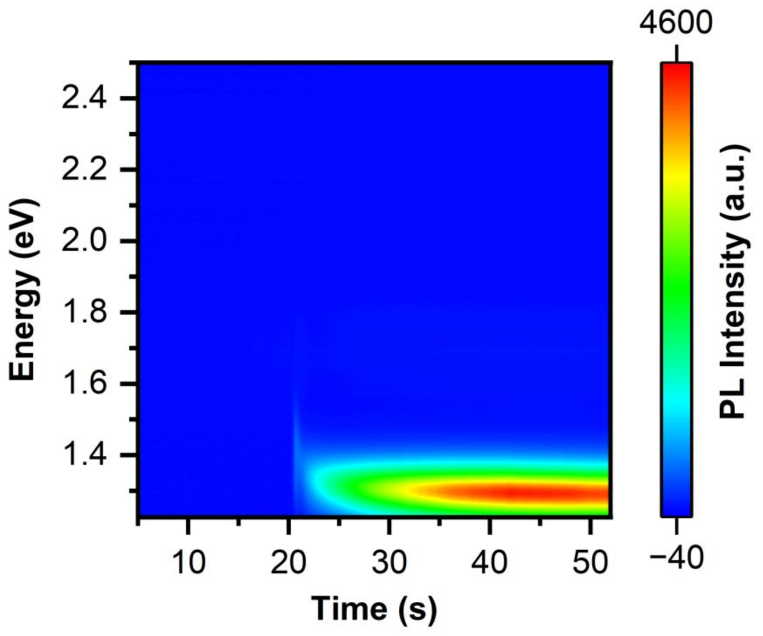

Figure S15. Contour plot of PL spectra for 10% GASCN film, ranging from 1.2 to 2.5 eV.

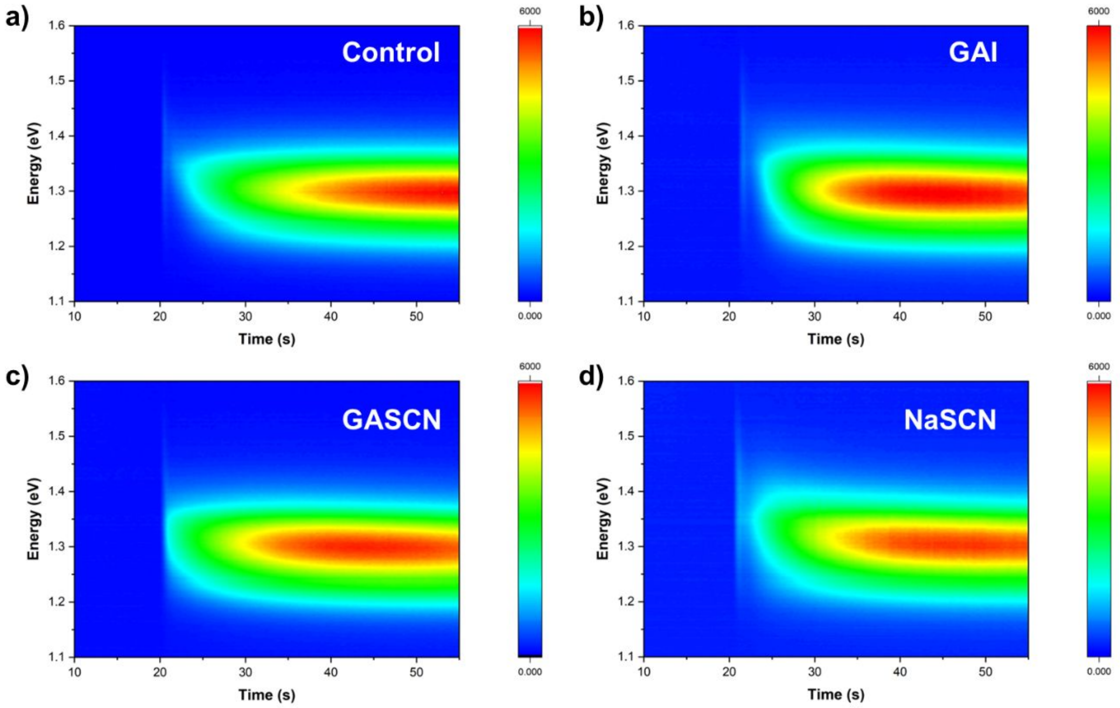

Figure S16. Contour plots of PL spectra for **a)** control (0% GASCN, without additive), **b)** 10% GAI, **c)** 10% GASCN, and **d)** 10% NaSCN films during spin-coating.

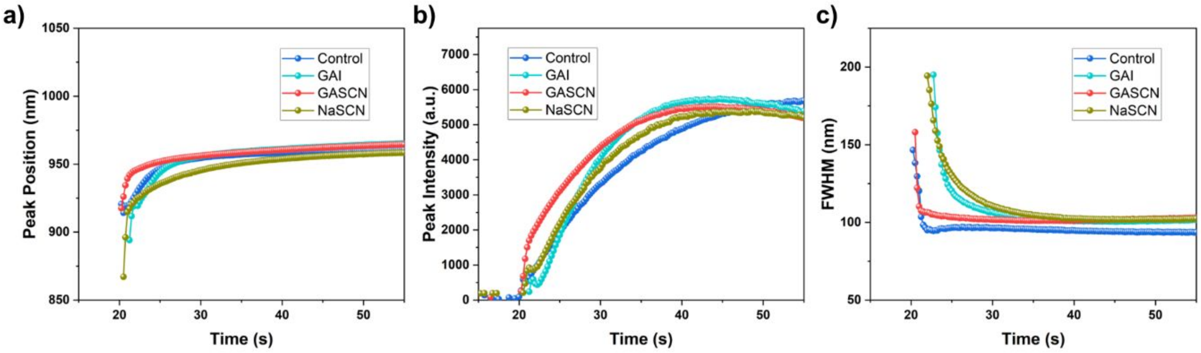

Figure S17. Peak information plots obtained from *in situ* PL measurements for **a)** peak position, **b)** peak intensity, and **c)** peak FWHM during spin-coating.

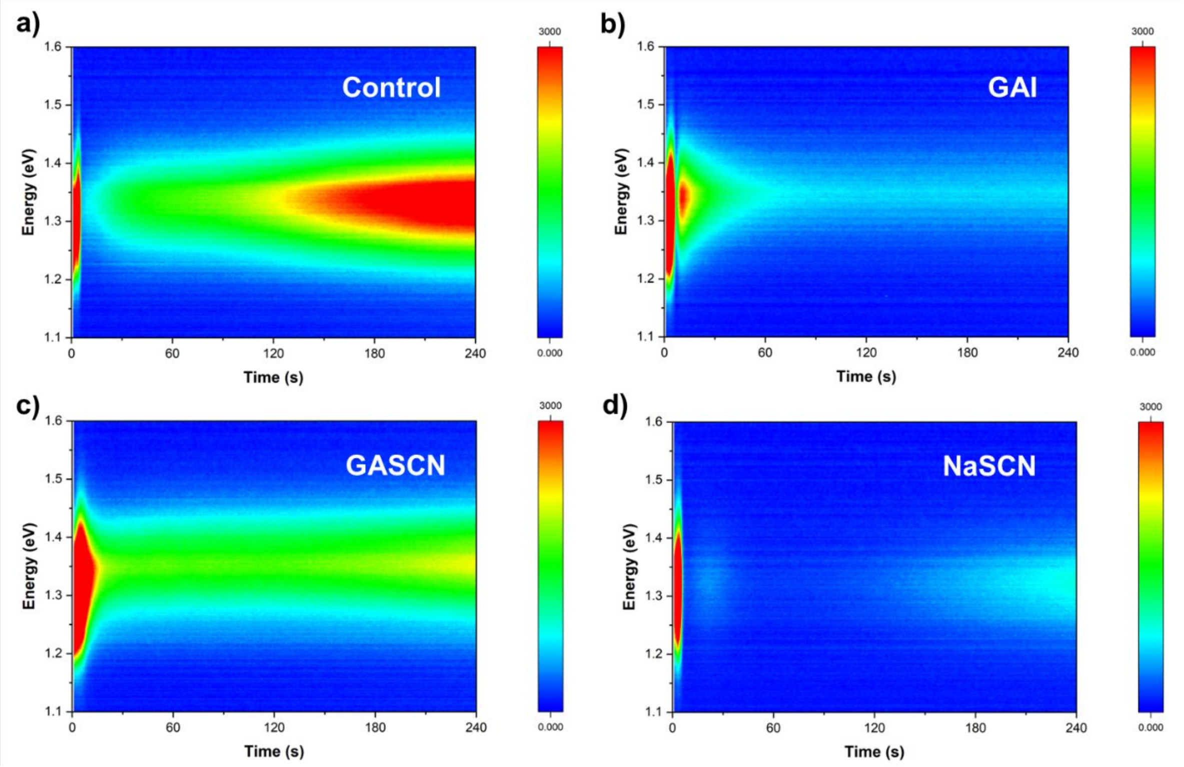

Figure S18. Contour plots of PL spectra for **a)** control, **b)** GAI, **c)** GASCN, and **d)** NaSCN films during annealing.

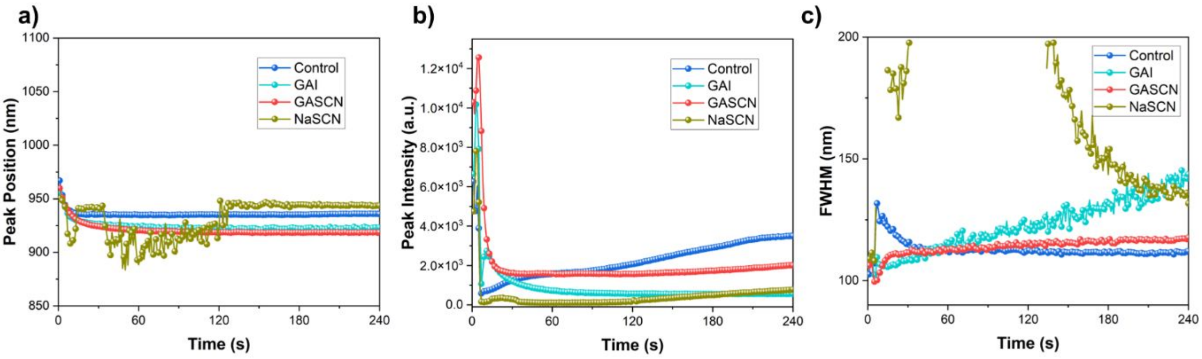

Figure S19. Peak information plots obtained from *in situ* PL measurements for **a)** peak position, **b)** peak intensity, and **c)** peak FWHM during annealing.

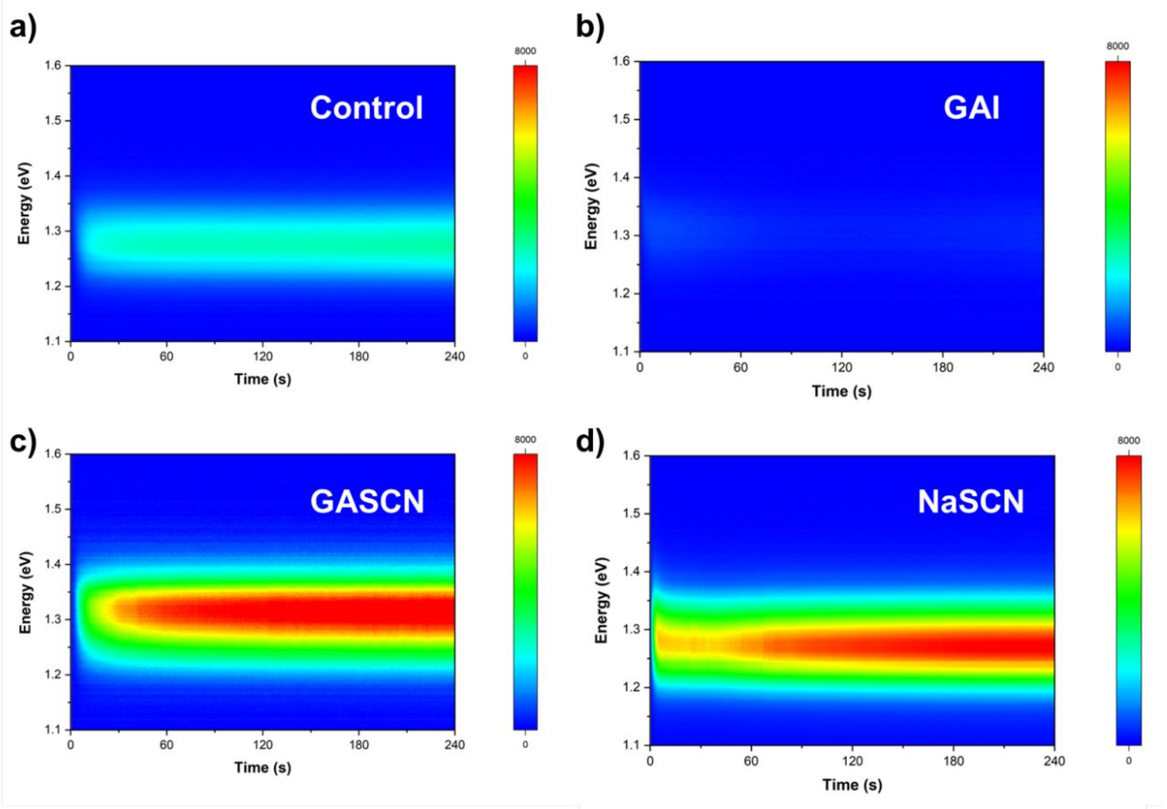

Figure S20. Contour plots of PL spectra for **a)** control, **b)** GAI, **c)** GASCN, and **d)** NaSCN films during cooldown.

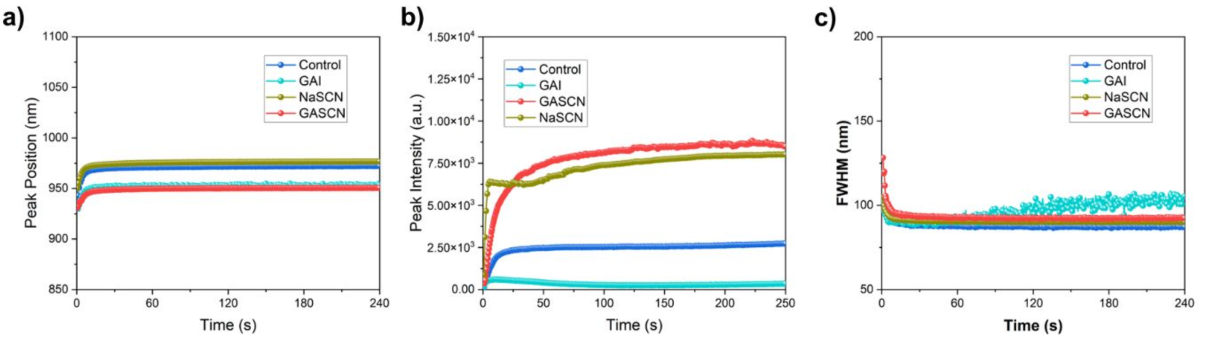

Figure S21. Peak information plots obtained from *in situ* PL measurements for **a)** peak position, **b)** peak intensity, and **c)** peak FWHM during cooldown.

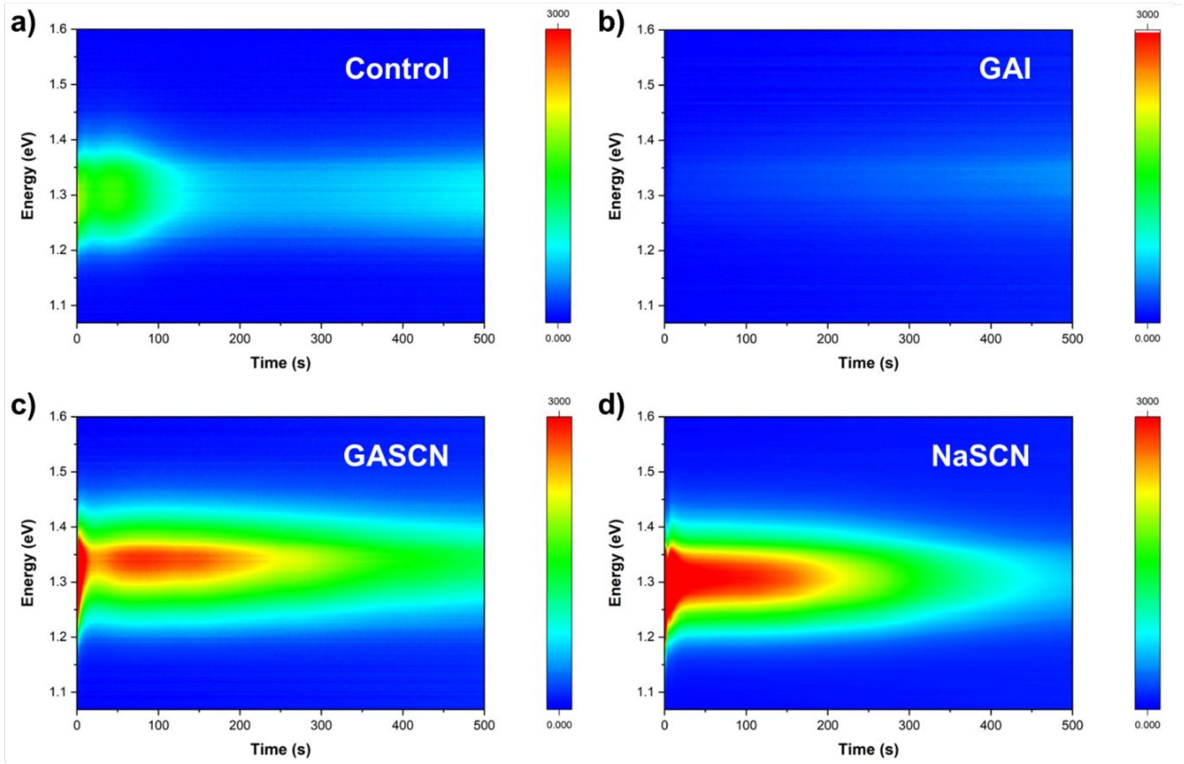

Figure S22. Contour plots of PL spectra for **a)** control, **b)** GAI, **c)** GASCN, and **d)** NaSCN films upon reheating at 75°C.

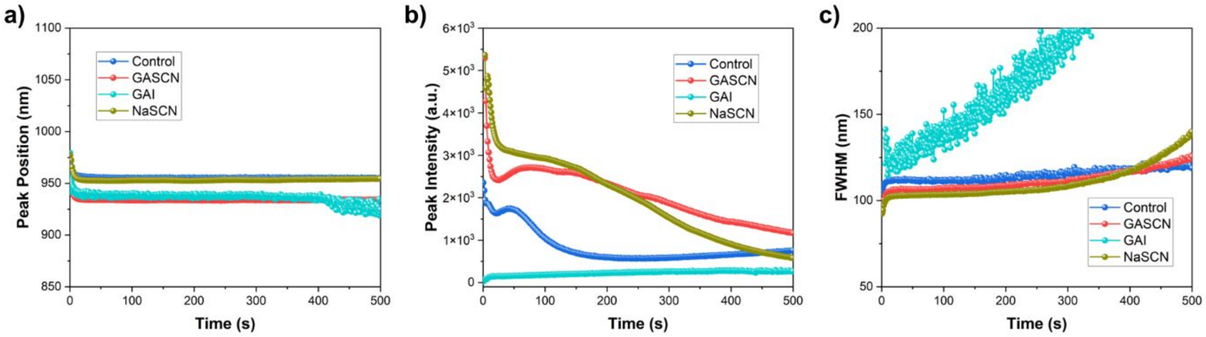

Figure S23. Peak information plots obtained from *in situ* PL measurements for **a)** peak position, **b)** peak intensity, and **c)** peak FWHM upon reheating at 75°C.

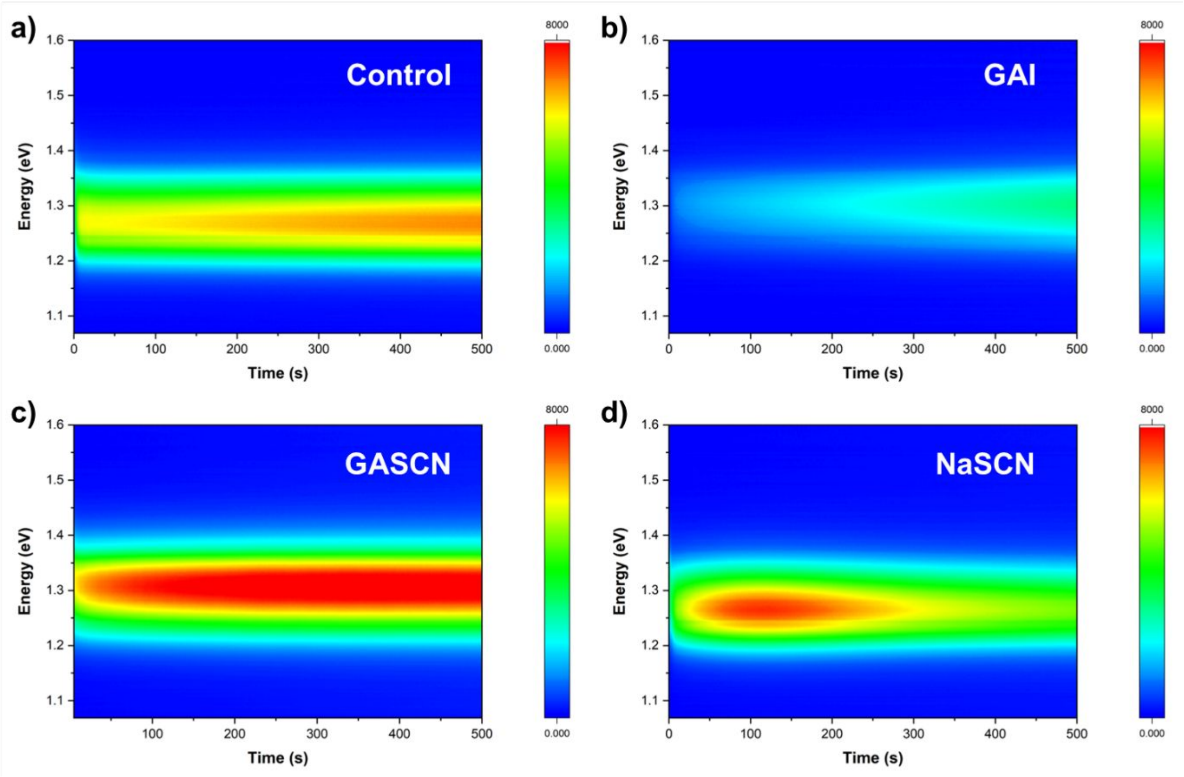

Figure S24. Contour plots of PL spectra for **a)** control, **b)** GAI, **c)** GASCN, and **d)** NaSCN films upon cooling down to room temperature (20-25℃) after reheating at 75°C.

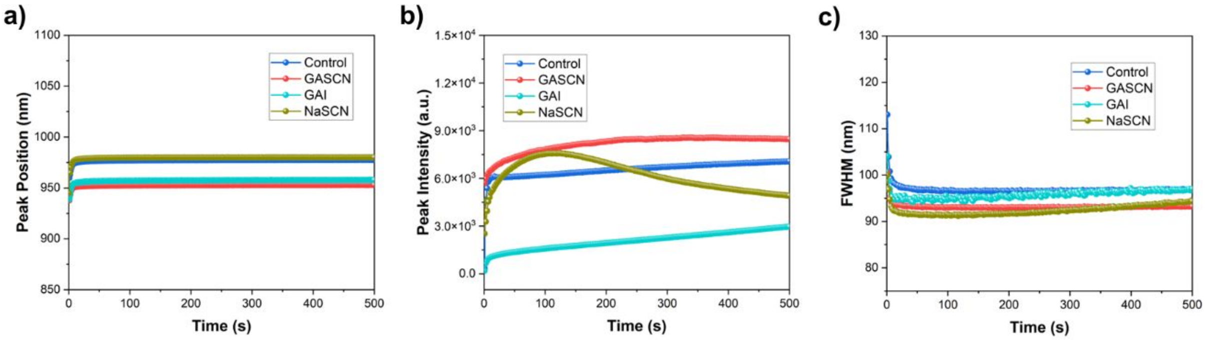

Figure S25. Peak information plots obtained from *in situ* PL measurements for **a)** peak position, **b)** peak intensity, and **c)** peak FWHM upon cooling down after reheating at 75°C.
